# Biomimetic Self‐Propelled Asymmetric Nanomotors for Cascade‐Targeted Treatment of Neurological Inflammation

**DOI:** 10.1002/advs.202310211

**Published:** 2024-03-09

**Authors:** Jiamin Ye, Yueyue Fan, Yaoguang She, Jiacheng Shi, Yiwen Yang, Xue Yuan, Ruiyan Li, Jingwen Han, Luntao Liu, Yong Kang, Xiaoyuan Ji

**Affiliations:** ^1^ Academy of Medical Engineering and Translational Medicine Medical College Tianjin University Tianjin 300072 China; ^2^ Department of General Surgery the First Medical Center Chinese People's Liberation Army General Hospital Beijing 100853 China; ^3^ Tianjin Key Laboratory of Radiation Medicine and Molecular Nuclear Medicine Institute of Radiation Medicine Chinese Academy of Medical Sciences and Peking Union Medical College Tianjin 100730 China; ^4^ Medical College Linyi University Linyi 276000 China

**Keywords:** anti‐inflammation, asymmetric, macrophage polarization, nanomotor, nanozyme, neurological inflammation

## Abstract

The precise targeted delivery of therapeutic agents to deep regions of the brain is crucial for the effective treatment of various neurological diseases. However, achieving this goal is challenging due to the presence of the blood‒brain barrier (BBB) and the complex anatomy of the brain. Here, a biomimetic self‐propelled nanomotor with cascade targeting capacity is developed for the treatment of neurological inflammatory diseases. The self‐propelled nanomotors are designed with biomimetic asymmetric structures with a mesoporous SiO_2_ head and multiple MnO_2_ tentacles. Macrophage membrane biomimetic modification endows nanomotors with inflammatory targeting and BBB penetration abilities The MnO_2_ agents catalyze the degradation of H_2_O_2_ into O_2_, not only by reducing brain inflammation but also by providing the driving force for deep brain penetration. Additionally, the mesoporous SiO_2_ head is loaded with curcumin, which actively regulates macrophage polarization from the M1 to the M2 phenotype. All in vitro cell, organoid model, and in vivo animal experiments confirmed the effectiveness of the biomimetic self‐propelled nanomotors in precise targeting, deep brain penetration, anti‐inflammatory, and nervous system function maintenance. Therefore, this study introduces a platform of biomimetic self‐propelled nanomotors with inflammation targeting ability and active deep penetration for the treatment of neurological inflammation diseases.

## Graphical abstract

A biomimetic drug‐loaded self‐propelled MnO_2_‐Au‐mSiO_2_@Cur nanomotor is developed with an asymmetric octopus‐type antenna structure that can target brain inflammation across the blood–brain barrier, realize deep penetration during inflammation, enhance the anti‐inflammatory effect and promote the polarization of microglia from the M1 to the M2 phenotype.

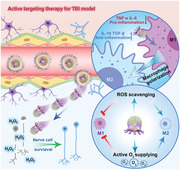

## 1 Introduction

Neurological diseases affecting the central nervous system (CNS) impose a significant global burden, impacting millions of individuals worldwide.^[^ ^1^ ^]^ These disorders encompass a wide range of conditions, including neurodegenerative diseases, neuropsychiatric disorders, neuroinflammatory conditions, and brain tumors.^[^ ^2^ ^]^ The consequences of these diseases extend beyond individual suffering, incurring substantial economic and societal costs.^[^ ^3^ ^]^ To develop effective therapeutic interventions, it is crucial to comprehend the underlying pathogenesis of these diseases. In particular, inflammation within the nervous system often accompanies the occurrence and progression of various neurological disorders and plays a pivotal role in their pathogenesis.^[^ ^4^ ^]^ It is well known that moderate levels of reactive oxygen species (ROS) participate in metabolic regulation and stress responses, supporting cellular adaptation to changing environments and stress. However, the overexpression of ROS within inflamed brain tissues not only damages biomolecules and structures but also triggers immune cells to produce excessive amounts of inflammatory factors.^[^ ^5^ ^]^ Consequently, effectively and rapidly mitigating the inflammatory response at the site of neural damage and maintaining the stability of the neuromicroenvironment through immune microenvironment regulation are primary considerations in neurological disease treatment.

The treatment of neurological disorders often requires the precise delivery of therapeutic agents to specific target regions within the brain. However, the barrier action of the blood–brain barrier (BBB) and the complex architecture of deep brain structures pose significant challenges to effective drug delivery.^[^ ^6^ ^]^ The BBB is composed of an intricate system of endothelial cells, pericytes, astrocytes, and other supporting cells that regulate the exchange of substances between the bloodstream and brain tissue.^[^ ^7^ ^]^ This barrier is crucial for maintaining homeostasis in the brain microenvironment and protecting it from harmful substances and toxins. However, this barrier also presents a significant challenge for delivering therapeutic agents to the brain, as many drugs are unable to cross this barrier and reach target cells.^[^ ^8^ ^]^ Furthermore, the complexity and inaccessibility of deep brain structures pose additional challenges for effective drug delivery, and conventional methods often fail to achieve optimal drug concentrations in these target regions while avoiding off‐target effects and systemic toxicity.^[^ ^9^ ^]^ Innovative strategies that combine BBB passage and deep brain permeation ability show great promise in overcoming these barriers and enabling precise drug delivery to specific deep brain structures.^[^ ^10^ ^]^ These strategies offer new hope for more effective treatment of neurological disorders.

Various approaches have been developed to overcome the challenges associated with drug delivery across the BBB.^[^ ^11^ ^]^ Physical methods, such as focused ultrasound and laser irradiation, have been explored for their ability to transiently disrupt the BBB and enhance drug penetration.^[^ ^12^ ^]^ Moreover, chemical and biochemical approaches, including liposome, nanoparticle, and prodrug formulations, have also shown promise in improving drug stability, solubility, and specificity.^[^ ^13^ ^]^ These approaches enhance the transport of drugs across the BBB and enable targeted delivery to specific brain regions. Additionally, recent studies have shown that certain immune cells can autonomously penetrate the BBB, particularly in response to brain inflammation.^[^ ^14^ ^]^ Interestingly, proteins such as CD44 and CD11b on the surface of macrophage membranes (MM) enable their passage across the BBB and accumulation in inflamed brain tissue.^[^ ^15^ ^]^ Therefore, utilizing macrophage‐modified nanoparticles allows active targeting of inflammatory sites within the brain. By employing these innovative strategies, researchers aim to improve drug delivery across the BBB and achieve precise targeting of therapeutic agents to specific brain regions, particularly those affected by inflammation in neurological disorders.

In recent years, artificial nanomotors have emerged as a promising research area with significant potential in biomedical applications.^[^ ^16^ ^]^ These nanomotors have the unique ability to propel themselves through various mechanisms, including chemical fuel, magnetic fields, or acoustic waves.^[^ ^17^ ^]^ This capability allows them to navigate through complex brain tissue and ultimately reach specific target sites. Among these nanomotors, self‐driven nanomotors, which can utilize overexpressed biomarkers present in the disease microenvironment, such as H_2_O_2_, as a source of fuel to power their own movement, are of particular interest.^[^ ^18^ ^]^ By converting these harmful biomarkers into their driving forces, these nanomotors can actively move within brain tissue.^[^ ^19^ ^]^ By harnessing the active movement capabilities of artificial nanomotors, researchers hope to design innovative strategies for delivering therapeutic agents to deeply damaged tissues within the brain.^[^ ^20^ ^]^ This approach has the potential to significantly enhance drug delivery efficiency and efficacy in neurological diseases. The development of artificial nanomotors with active motion capacity represents a novel and exciting direction in the field of neurology and holds promise for enhancing treatment outcomes.

**Figure 1:**
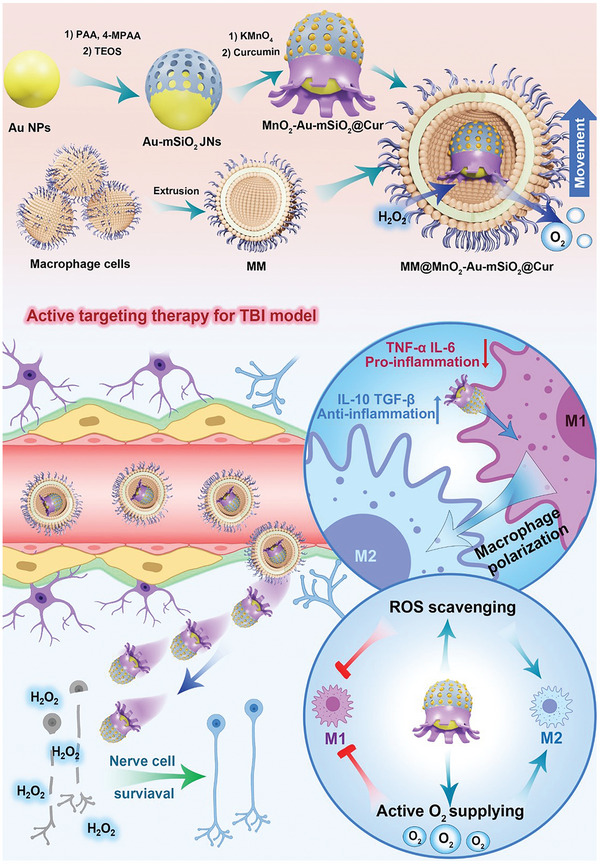
Schematic illustration of the fabrication process of MM@MnO_2_‐Au‐mSiO_2_@Cur and cascade‐targeting anti‐inflammatory therapy for TBI.

In this study, we proposed a cascade deep brain delivery strategy utilizing biomimetic Cur‐loaded self‐propelled nanomotors (MM@MnO_2_‐Au‐mSiO_2_@Cur) for targeted anti‐inflammatory therapy (**Figure** **1**). By mimicking the surface characteristics of macrophages, biomimetic nanomotors can actively accumulate in regions of inflammation through the flow of blood without being hindered by the BBB. The MnO_2_‐Au‐mSiO_2_ structure contains partially encapsulated MnO_2_ antennas, which can convert ROS into oxygen bubbles, and these bubbles act as a driving force for the autonomous motion of the nanomotors. Moreover, the produced oxygen quickly diffuses throughout the entire brain instead of accumulating in a particular area. Furthermore, the generated oxygen also helps alleviate hypoxia, enhancing the efficacy of anti‐inflammatory treatment. Curcumin (Cur), an anti‐inflammatory drug, was loaded into the mesopores of the MnO_2_‐Au‐mSiO_2_ structure. Cur effectively alleviated inflammation and regulated the polarization of macrophages and microglia from a proinflammatory (M1) phenotype to an anti‐inflammatory (M2) phenotype, which contributed to the treatment of neuroinflammatory disorders. To validate the effectiveness of these self‐propelled biomimetic nanomotors, animal traumatic brain injury (TBI) models were generated. In vitro cell and organoid models, as well as in vivo animal experiments, confirmed the accurate targeting of brain injury sites by biomimetic nanomotors through cascade targeting. Additionally, the catalytic chemical reactions of the nanomotors provide deep penetration into brain tissue. Overall, this study presents a novel perspective on the design of biomimetic drug‐loaded nanomotors and their potential application in anti‐inflammatory treatments for neurological disorders involving inflammation.

## 2 Results and Discussion

### 2.1 Preparation and Characterization of the Biomimetic Nanomotors

**Figure 2:**
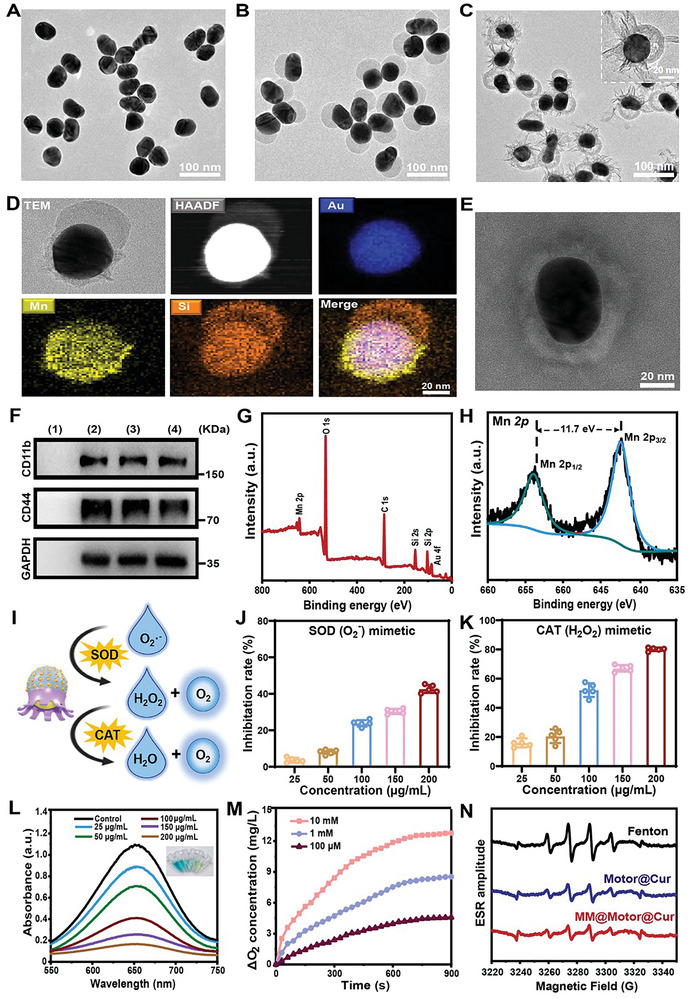
Preparation and characterization of MM@MnO_2_‐Au‐mSiO_2_@Cur. TEM images of A) Au NPs, B) Au‐mSiO_2_ JNs, and C) MnO_2_‐Au‐mSiO_2_. D) HAADF image and corresponding mapping image of MnO_2_‐Au‐mSiO_2_. E) TEM image of MM@MnO_2_‐Au‐mSiO_2_@Cur. F) Western blot analysis of different samples. G) Full XPS spectrum of MnO_2_‐Au‐mSiO_2_@Cur. H) High‐resolution XPS spectrum of Mn 2p. I) Schematic of the CAT‐ and SOD‐like capacities of MnO_2_‐Au‐mSiO_2_@Cur. J) SOD and K) CAT mimetic activities of different concentrations of MnO_2_‐Au‐mSiO_2_@Cur. L) UV‒vis spectra of TMB solution in the presence of H_2_O_2_ and different concentrations of MnO_2_‐Au‐mSiO_2_@Cur. M) O_2_ generation in the presence of different concentrations of H_2_O_2_ in the presence of MnO_2_‐Au‐mSiO_2_@Cur (100 µg mL^−1^). N) ESR spectra of different treatments using DMPO as a spin trap agent. The results are presented as the mean ± SD.

The synthesis of MnO_2_‐Au‐mSiO_2_ nanomotors was performed via a wet‐chemistry method with an asymmetric structure. First, high‐quality Au nanoparticles (≈50 nm) were synthesized through citrate reduction^[^ ^21^ ^]^ (**Figure** **2**A). Then, SiO_2_ was selectively deposited on one side of the Au nanoparticles, forming Janus Au‐SiO_2_ nanoparticles through ligand competition between 4‐mercaptophenylacetic acid (4‐MPAA) and poly(acrylic acid) (PAA)^[^ ^21^, ^22^ ^]^ (Figure 2B). Next, we employed the “surface protected etching” strategy to create a mesoporous SiO_2_ (mSiO_2_) structure (Figure S1, Supporting Information). Potassium permanganate (KMnO_4_) was added and reduced by the ligands on the exposed side of the Au nanoparticles, resulting in the formation of MnO_2_‐Au‐mSiO_2_ Janus nanomotors. Scanning electron microscopy (SEM) and transmission electron microscopy (TEM) were used to visualize the asymmetry of the structure of MnO_2_‐Au‐mSiO_2_ with octopus‐like tentacles (Figure 2C; Figures S2 and S3, Supporting Information). High‐angle annular dark‐field (HADDF) imaging and elemental mapping further confirmed the asymmetric structure of the MnO_2_‐Au‐mSiO_2_ Janus nanomotors (Figure 2D). Additionally, we prepared macrophage membrane (MM) vesicles using a liposome extruder (Figure S4, Supporting Information). To achieve targeted delivery to alleviate TBI‐related inflammation, the MM vesicles and nanomotors were coextruded multiple times through a liposome extruder, resulting in the formation of biomimetic MM‐coated nanomotors (Figure 2E). The successful encapsulation of MM vesicles on the outer surface of the nanomotors was further confirmed through Western blot analysis (Figure 2F). These experimental techniques and results provide comprehensive evidence for the synthesis and characterization of MnO_2_‐Au‐mSiO_2_ nanomotors and successful coating with MM vesicles, demonstrating their potential for targeted therapy for TBI‐related inflammation.

In addition, we analyzed the pore size distribution and specific surface area of MnO_2_‐Au‐mSiO_2_. The BET surface area was measured to be 478.27 ± 2.99 m^2^ g^−1^, and the pore size was ≈3.6 nm (Figure S5, Supporting Information). Moreover, the incorporation of a mesoporous mSiO_2_ shell enhanced the drug loading capacity of MnO_2_‐Au‐mSiO_2_, resulting in the formation of Cur‐loaded MM vesicles coated with MnO_2_‐Au‐mSiO_2_ (MM@MnO_2_‐Au‐mSiO_2_@Cur). The successful loading of Cur into the mesoporous shell of MM@MnO_2_‐Au‐mSiO_2_ was confirmed by the observation of an ultraviolet‒visible (UV‒vis) absorption peak at ≈410 nm (Figure S6, Supporting Information). The particle size of MM@MnO_2_‐Au‐mSiO_2_@Cur was ≈20 nm larger than that of MnO_2_‐Au‐mSiO_2_@Cur, and the zeta potential decreased from −2.3 to −10.3 mV, providing evidence for the successful fabrication of MM vesicles on the surface of MnO_2_‐Au‐mSiO_2_@Cur (Figures S7 and S8, Supporting Information). Furthermore, the chemical composition and crystal structure of MnO_2_‐Au‐mSiO_2_@Cur were analyzed using X‐ray photoelectron spectroscopy (XPS) and X‐ray diffraction (XRD), revealing the presence of Mn, O, C, Si, and Au as the main elements in MnO_2_‐Au‐mSiO_2_@Cur (Figure 2G,H; Figures S9 and S10, Supporting Information). Notably, the spin‐orbit peak splitting distance between the Mn 2p_3/2_ and Mn 2p_1/2_ peaks was measured to be 11.7 eV, further confirming the existence of the MnO_2_ nanozyme. These results provide conclusive evidence for the successful fabrication of MM@MnO_2_‐Au‐mSiO_2_@Cur.

### 2.2 Enzyme‐Like Activity Property Analysis

The Cur‐loaded nanomotors exhibited nanoenzymatic properties through their catalase (CAT) and superoxide dismutase (SOD) enzyme‐like activities (Figure 2I). Initially, the SOD‐mimicking activity of MnO_2_‐Au‐mSiO_2_ was evaluated using an SOD kit with WST‐8. The presence of MnO_2_‐Au‐mSiO_2_ nanomotors significantly facilitated the effective clearance of O_2_·^−^ (Figure S11, Supporting Information). Surprisingly, after loading with Cur, the inhibition rate of O_2_·^−^ reached 64.6%, indicating an ≈22% improvement in clearance rates compared to MnO_2_‐Au‐mSiO_2_ alone (Figure 2J). Similar trends were observed for CAT enzyme activation (Figure 2K; Figure S12, Supporting Information).

To investigate the catalytic consumption of H_2_O_2_ by MnO_2_‐Au‐mSiO_2_@Cur, we employed 3,3′,5,5′‐tetramethylbenzidine (TMB) as an indicator. TMB undergoes oxidation to form oxidized TMB (oxTMB) in the presence of H_2_O_2_ and Fe^2+^ through the Fenton reaction.^[^ ^23^ ^]^ After incubation with different concentrations of MnO_2_‐Au‐mSiO_2_@Cur, there was a significant decrease in UV absorption at 652 nm, indicating the consumption of H_2_O_2_ by MnO_2_‐Au‐mSiO_2_@Cur (Figure 2L). Similar results were observed at a wavelength of 450 nm after the addition of a stopping solution (Figure S13, Supporting Information). Moreover, the decomposition of O_2_·^−^ and H_2_O_2_ results in the production of oxygen bubbles, which contributes to the self‐propelled propulsion of the nanomotors. Additionally, a significant number of bubbles was observed when H_2_O_2_ was mixed with MnO_2_‐Au‐mSiO_2_@Cur (Figure S14, Supporting Information). The generation of oxygen induced by the mixture of MnO_2_‐Au‐mSiO_2_@Cur and H_2_O_2_ was further confirmed using a dissolved oxygen meter (Figure 2M). As shown in Figure 2M, when the concentration of H_2_O_2_ reached 1 mm, the oxygen content generated by the biomimetic nanomotors was ≈8 mg L^−1^ within 900 s. Furthermore, MnO_2_‐Au‐mSiO_2_@Cur was confirmed to be an effective scavenger of ROS. Electron spin resonance (ESR) analysis was applied to evaluate the ROS scavenging activities of MnO_2_‐Au‐mSiO_2_@Cur using ·OH generated from the reaction between Fe^2+^ and H_2_O_2_ via the Fenton reaction as a representative ROS model. The ESR spectrum exhibited a characteristic peak corresponding to 5‐dimethyl‐1‐pyrroline‐N‐oxide (DMPO) after the Fenton reaction, indicating successful ·OH generation (Figure 2N). Upon the addition of MnO_2_‐Au‐mSiO_2_@Cur, the peak intensity noticeably decreased. Similarly, when MM‐coated MnO_2_‐Au‐mSiO_2_@Cur was added, a reduction in peak intensity was observed, demonstrating the excellent ROS scavenging ability of MM@MnO_2_‐Au‐mSiO_2_@Cur. These findings collectively suggest that MnO_2_‐Au‐mSiO_2_@Cur possesses remarkable SOD and CAT enzyme‐like activities. Furthermore, the addition of Cur enhances the enzyme‐like activities of the nanomotors and enhances the ability of the NPs to clear ROS.

### 2.3 Autonomous Propulsion Movement Performance

**Figure 3:**
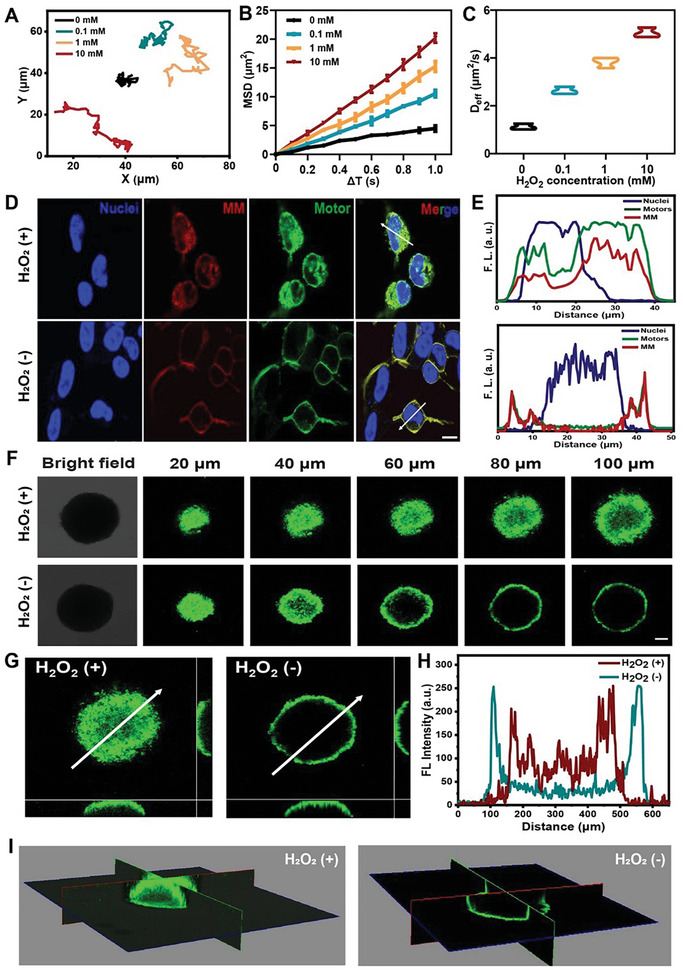
The autonomous movement behavior of the MnO_2_‐Au‐mSiO_2_@Cur nanomotors. A) Motion trajectories of MnO_2_‐Au‐mSiO_2_@Cur nanoparticles at different concentrations of H_2_O_2_. B) MSD plots and C) D_eff_ of MnO_2_‐Au‐mSiO_2_@Cur nanoparticles treated with different concentrations of H_2_O_2_ (*n* = 3). D) Cellular uptake and E) relative fluorescence spectra of FITC‐ and DiI‐labeled MM@MnO_2_‐Au‐mSiO_2_@Cur NPs with or without H_2_O_2_ treatment. Scale bar, 100 µm. F) CLSM images of 3D SH‐SY5Y spheroids treated with MnO_2_‐Au‐mSiO_2_@Cur (with or without H_2_O_2_). Scale bar, 100 µm. G) The section view and H) the corresponding mean fluorescence intensity with or without H_2_O_2_. I) 3D scan view of 3D cell spheroids after different treatments. The results are presented as the mean ± SD.

Taking advantage of their efficient catalase (CAT) enzyme activity, MnO_2_‐Au‐mSiO_2_ nanomotors, which have octopus‐type asymmetric structures, generate oxygen bubbles by catalyzing H_2_O_2_. Based on this principle, we investigated the motion performance of the nanomotors in the presence of H_2_O_2_ as fuel. The representative tracking trajectories of the MnO_2_‐Au‐mSiO_2_ nanomotors at different concentrations of H_2_O_2_ are presented in **Figure** **3**A and Figure S15 (Supporting Information). The longest motion trajectory was observed when the nanomotors were incubated with H_2_O_2_. As the concentration of H_2_O_2_ increased, both the effective diffusion coefficient (D_eff_) and mean square displacement (MSD) of the nanomotors significantly increased (Figure 3B,C). This finding confirmed the excellent self‐propelled performance of the nanomotors driven by H_2_O_2_ fuel.

Encouraged by these findings, we further investigated the intracellular motion and penetration capacity of the nanomotors. Efficient uptake by cells is necessary for subsequent movements. Therefore, we first assessed the uptake of the nanomotors by SH‐SY5Y cells. To better visualize the intracellular movements of the MM@MnO_2_‐Au‐mSiO_2_ nanomotors, we labeled MnO_2_‐Au‐mSiO_2_ with FITC dye and labeled the MM component with DiI dye. SH‐SY5Y cells were incubated with the MM@MnO_2_‐Au‐mSiO_2_ nanomotors for 2 h, with or without H_2_O_2_ stimulation. Strong FITC and DiI signals were observed under H_2_O_2_ treatment, indicating more efficient uptake of the nanomotors by SH‐SY5Y cells after H_2_O_2_ treatment (Figure 3D,E). The active movement capability of nanomotors allows them to be rapidly taken up by cells and facilitates their escape from lysosomes upon entering the cells, thereby enhancing their therapeutic efficacy.^[^ ^24^ ^]^ To further investigate the intracellular distribution of the nanomotors, which is crucial for their transport and effectiveness, we examined their distribution after they entered SH‐SY5Y cells. As expected, the FITC‐labeled nanomotors treated with H_2_O_2_ escaped from the lysosome, while the majority of the nanomotors not treated with H_2_O_2_ exhibited a high colocalization rate in the lysosome (Figure S16, Supporting Information). These results indicate that the movement of the nanomotors can facilitate lysosomal escape and continuous accumulation within cells.

Furthermore, to evaluate the self‐driven motion ability of the MnO_2_‐Au‐mSiO_2_ nanomotors, we established 3D SH‐SY5Y cell spheroid models. After incubation with the nanomotors, confocal laser scanning microscopy (CLSM) was used to capture images of different sections of the spheroids incubated with the MnO_2_‐Au‐mSiO_2_ nanomotors, with or without additional H_2_O_2_ treatment. As shown in Figure 3F, the sections of cell spheroids in the nanomotor group treated with H_2_O_2_ exhibited more prominent fluorescence than did those in the group not treated with H_2_O_2_. Moreover, the increase in fluorescence intensity across the 80 nm region confirmed the high fluorescence signal in the nanomotor group after H_2_O_2_ treatment (Figure 3G,H). 3D images of the cell spheroids further demonstrated the high penetration capacity of the nanomotors after H_2_O_2_ treatment inside the spheroids (Figure 3I). Moreover, no significant fluorescence was observed within the 3D spheroids of the Au‐mSiO_2_ nanoparticle‐treated group after stimulation with H_2_O_2_ (Figure S17, Supporting Information), which demonstrated the crucial role of asymmetrically modified MnO_2_ in driving the self‐propelled motion of the MnO_2_‐Au‐mSiO_2_ nanomotor. These results confirmed that self‐propelled nanomotors driven by H_2_O_2_ fuel can achieve rapid intracellular motion and high intracellular permeability.

### 2.4 BBB Penetration Capacity of the Biomimetic Nanomotors

**Figure 4:**
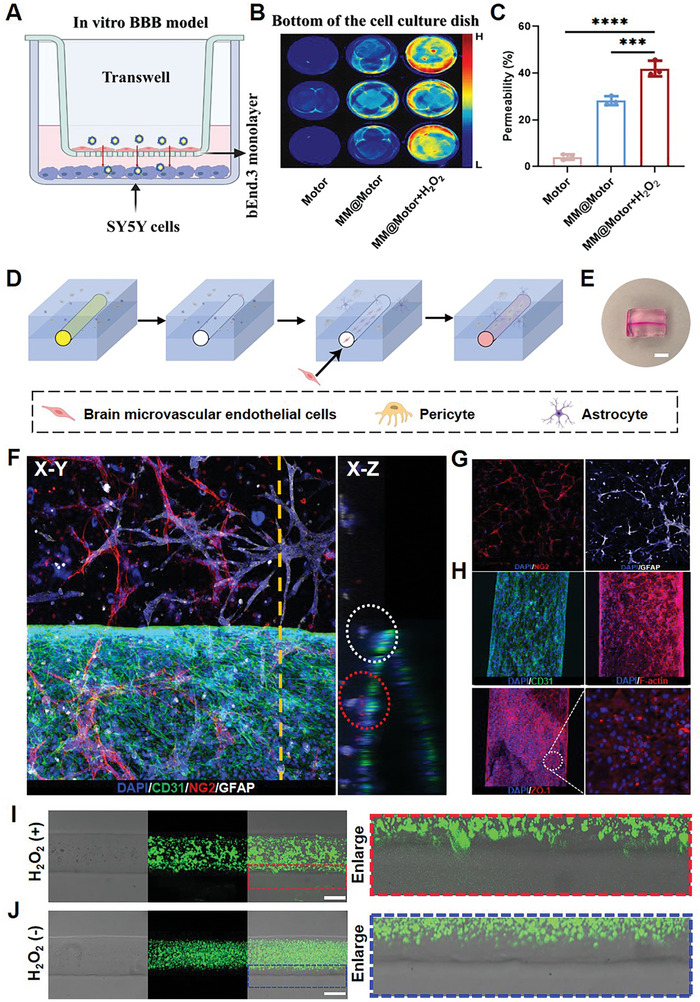
The capacity to actively cross the BBB in vitro. A) Schematic showing the construction of the in vitro BBB model. B) Fluorescence images of the lower chamber after different treatments. C) BBB transport efficiency after different treatments. D) Schematic showing the protocol used to construct the 3D BBB organoid model. E) Digital photograph of the 3D BBB organoid model. Scale = 2 mm. F) CLSM images showing the X‐Y plane and the cross section of the X‐Z plane at the yellow dashed line of brain microvascular networks, which are composed of brain microvascular endothelial cells (CD31, green), pericytes (NG2, red), and astrocytes (GFAP, white). G) CLSM images of microvascular markers. The capacity of the FITC‐labeled motor to establish a 3D BBB organoid model H) with or without H2O2 treatment I). Scale bar, 200 µm. All the results are presented as the mean ± SD (*n* = 3). A statistically significant difference was assessed by one‐way ANOVA (***P < 0.001, ****P < 0.0001).

To investigate the ability of MM@MnO_2_‐Au‐mSiO_2_ to cross the BBB and target neuronal cells, an in vitro BBB model was built using Transwell chambers (**Figure** **4**A). Indocyanine green (ICG)‐labeled MnO_2_‐Au‐mSiO_2_ and MM@MnO_2_‐Au‐mSiO_2_ were added to the upper chamber of the Transwell system and incubated for 4 h with or without additional H_2_O_2_ stimulation. Fluorescence images captured by an in vivo imaging system (IVIS) showed efficient enrichment of MM@MnO_2_‐Au‐mSiO_2_ in the lower chamber under H_2_O_2_ stimulation, indicating targeted delivery to SH‐SY5Y cells across the BBB barrier (Figure 4B). Additionally, the permeability of the nanomotors from the upper chamber to the lower chamber was measured via ICP‐MS analysis. MM@MnO_2_‐Au‐mSiO_2_ exhibited an ≈40% increase in permeability in response to H_2_O_2_ treatment compared to that in the MnO_2_‐Au‐mSiO_2_ group, indicating improved BBB permeability due to the inflammation‐targeting properties of MM and the self‐propelled motion of the nanomotors (Figure 4C).

To further examine the ability of MM@MnO_2_‐Au‐mSiO_2_ to traverse the BBB, we utilized 3D bioprinting technology to construct BBB organoids that replicated the physiological structure and barrier function (Figure 4D,E). Vascular endothelial cells formed a dense monolayer tubular structure, and peripheral cells aggregated around the blood vessels, covering their surface. CLSM images of the X‐Z plane showed that the foot processes of astrocytes extended to the vascular wall and formed close connections with endothelial cells and pericytes, indicating the development of important BBB components (Figure 4F). Furthermore, 3D scanning images clearly revealed the interaction between BBB‐specific junction proteins and basal proteins, which maintained the structure and function of the BBB (Figure 4G). FITC‐labeled nanomotors were added to the microtubular structure in the presence of H_2_O_2_ to simulate an inflammatory microenvironment. CLSM images revealed that more fluorescent signals traveled through the tubes to the outside of the GelMA matrix, indicating the ability of MM@MnO_2_‐Au‐mSiO_2_ to traverse the BBB under inflammatory conditions (Figure 4H). In contrast, there were no obvious signals on the outside of the microtubular structure without additional H_2_O_2_ (Figure 4I). These results demonstrated that MM biomimetic‐modified nanomotors powered by H_2_O_2_ fuel have the potential to effectively cross the BBB and reach inflammatory sites.

The mechanisms by which biomimetic macrophage membrane‐modified nanoparticles cross the BBB and enter sites of brain inflammation are as follows. The inflammatory response in inflamed brain tissue leads to an increase in cell adhesion molecules (CAMs), such as intercellular adhesion molecule‐1 (ICAM‐1) and P‐selectin, which are overexpressed on the luminal side of vascular endothelial cells.^[^ ^25^ ^]^ These molecules facilitate the adhesion of immune cells, such as macrophages, to the endothelial cells of the BBB and promote their infiltration into the lesion site, recruiting them regardless of the permeability of the BBB. In addition, during the inflammatory process, various chemical factors, such as cytokines and chemokines, are released by inflammatory cells and tissues. These factors can attract immune cells, including macrophages, to migrate toward sites of inflammation, which are not disrupted by the BBB.^[^ ^15b^ ^]^ Finally, brain inflammation destroys the structural and functional integrity of the BBB, increases the permeability of the BBB,^[^ ^2c^ ^]^ and further facilitates the passage of nanoparticles wrapped in macrophage membranes across the BBB into inflammatory sites in brain tissue. Therefore, biomimetic macrophage membranes derived from macrophage modification on the surface of self‐propelled nanomotors can enable them to move across the BBB and specifically target sites of brain inflammation for more effective treatment. After targeting the inflammatory sites in the central nervous system, biomimetic macrophage membrane‐modified nanomotors entered the inflammatory cells through endocytosis. Subsequently, the modified macrophage membrane may interact with lysosomes, triggering degradation of the macrophage membrane.^[^ ^14e^ ^]^ This process leads to the gradual disappearance or disruption of the macrophage membrane, resulting in the release of the drug‐loaded nanomotors.

### 2.5 ROS Scavenging and Neuroprotective Effects

**Figure 5:**
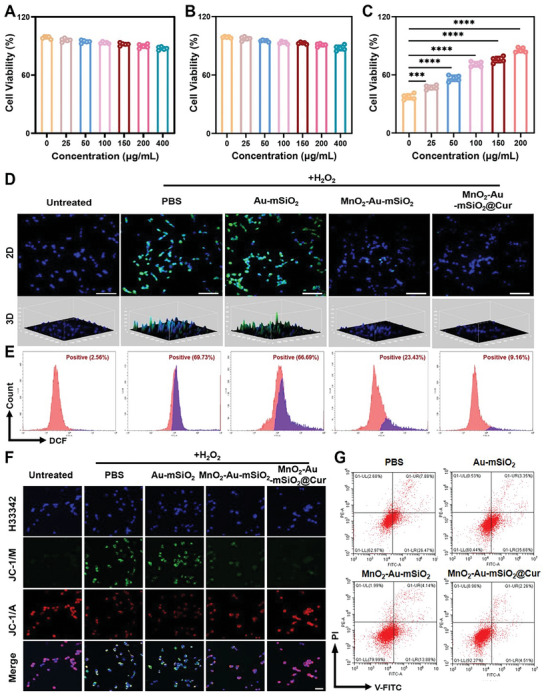
Neuroprotective effect of the MnO_2_‐Au‐mSiO_2_@Cur nanomotor. Cell viability of A) SH‐SY5Y and B) BV2 cells after treatment with different concentrations of MnO_2_‐Au‐mSiO_2_@Cur. C) SH‐SY5Y cell viability was determined by a CCK8 assay with different concentrations of MnO_2_‐Au‐mSiO_2_@Cur after H_2_O_2_ treatment (*n* = 5). D) Fluorescence images and E) flow cytometry (FCM) analysis of cellular ROS levels after different treatments. Scale bar, 100 µm. F) CLSM images of changes in the mitochondrial membrane potential after various treatments were analyzed via JC‐1 staining. Scale bar, 50 µm. G) FCM analysis of the percentage of apoptotic cells after different treatments in Annexin V‐FITC/PI‐stained SH‐SY5Y cells. All the results are presented as the mean ± SD. A statistically significant difference was assessed by one‐way ANOVA (****P < 0.0001).

Motivated by the excellent CAT and SOD enzyme‐like activities exhibited by the nanomotors, we subsequently investigated their ability to scavenge intracellular ROS and exert neuroprotective effects. The potential cytotoxicity of MnO_2_‐Au‐mSiO_2_@Cur was evaluated using the CCK‐8 assay, which demonstrated limited cytotoxicity, even at concentrations as high as 400 µg mL^−1^, to both SH‐SY5Y and BV2 nerve cells, indicating the excellent biocompatibility of MnO_2_‐Au‐mSiO_2_@Cur (**Figure** **5**A,B). We then incubated SH‐SY5Y cells with different concentrations of MnO_2_‐Au‐mSiO_2_@Cur after H_2_O_2_ stimulation to investigate the viability of these cells against H_2_O_2_‐induced cell apoptosis. Figure 5C shows that the viability of SH‐SY5Y cells incubated with H_2_O_2_ was only 37%, while the survival rate significantly improved in a dose‐dependent manner after incubation with different concentrations of MnO_2_‐Au‐mSiO_2_@Cur, indicating the neuroprotective effects of MnO_2_‐Au‐mSiO_2_@Cur. This result not only proves that the nanomotors maintain cell activity by effectively removing H_2_O_2_ but also verifies that the moderate amount of oxygen produced by the nanomotors does not cause toxicity to the cells.

Next, we investigated the ROS scavenging capacities of MnO_2_‐Au‐mSiO_2_@Cur using a DCFH‐DA kit. SH‐SY5Y and BV2 cells were stimulated with H_2_O_2_ to produce ROS, which were used as a positive control. DCFH‐DA staining revealed that the intensity of the green fluorescent signals decreased significantly after treatment with MnO_2_‐Au‐mSiO_2_@Cur (Figure 5D; Figure S18, Supporting Information). The flow cytometry (FCM) results further confirmed the sharp decrease in DCF signals after treatment with MnO_2_‐Au‐mSiO_2_@Cur, demonstrating the excellent intracellular ROS scavenging capacity of MnO_2_‐Au‐mSiO_2_@Cur (Figure 5E; Figure S19, Supporting Information). To investigate the neuroprotective effect of MnO_2_‐Au‐mSiO_2_@Cur on SH‐SY5Y cells, a mitochondrial membrane potential dye (JC‐1) was used. CLSM images revealed that treatment with only H_2_O_2_ caused obvious mitochondrial damage, while SH‐SY5Y cells treated with H_2_O_2_ and MnO_2_‐Au‐mSiO_2_@Cur exhibited a negligible decrease in the mitochondrial membrane potential (Figure 5F). Cell apoptosis was further verified by the Annexin V‐FITC/PI assay, which was consistent with the CLSM results (Figure 5G; Figures S20 and S21, Supporting Information). These results suggest that MnO_2_‐Au‐mSiO_2_@Cur can effectively scavenge intracellular ROS and simultaneously rescue damaged neurons induced by ROS.

### 2.6 Intracellular Oxygen Generation and Microglial Polarization In Vitro

**Figure 6:**
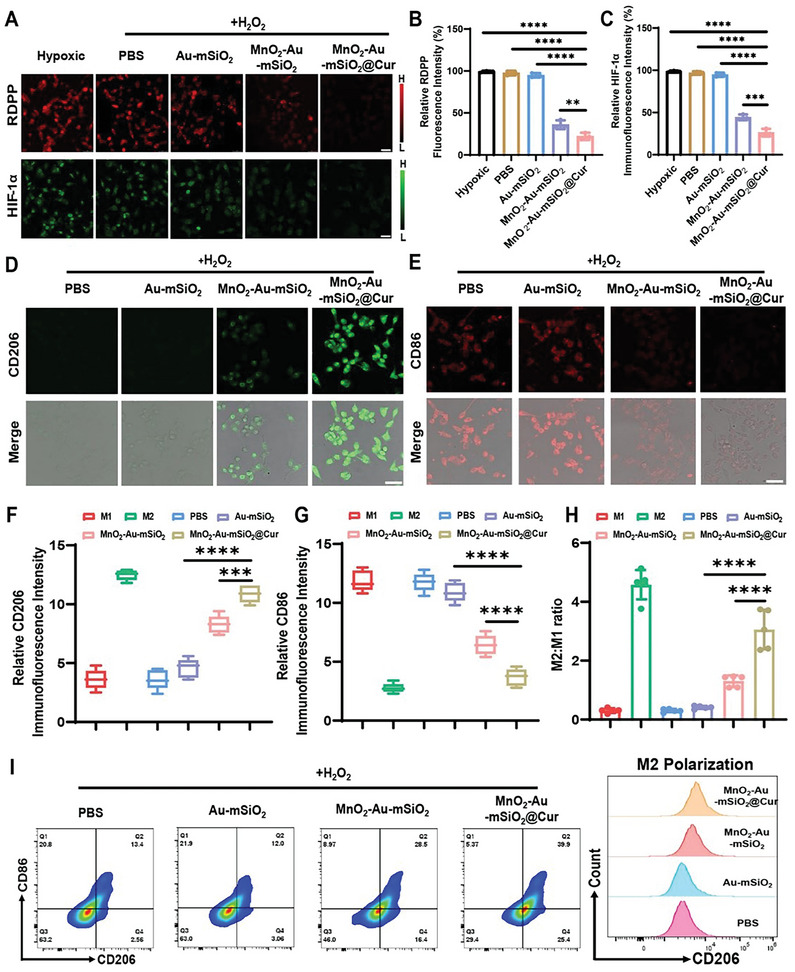
Regulation of microglial polarization of M1 to M2 by MnO_2_‐Au‐mSiO_2_@Cur nanoparticles. A) RDPP and HIF‐1α staining of BV2 cells after different treatments. Scale bar, 50 µm. The corresponding fluorescence intensities of B) RDPP and C) HIF‐1α are shown. CLSM images of microglia immunostained with D) CD206 (M2 marker) and E) CD86 (M1 marker). Scale bar, 50 µm. The relative F) CD206 and G) CD86 immunofluorescence intensities in BV2 cells after different treatments. H) Relative quantification of the M2/M1 ratio (*n* = 5). I) FCM analysis of microglial polarization after different treatments. All the results are presented as the mean ± SD. Significant differences were assessed by one‐way ANOVA (**P < 0.01, ***P < 0.001, ****P < 0.0001).

Macrophages and microglia, which are crucial immune cells in the brain, exhibit high plasticity after TBI and play key roles in regulating neuroinflammation and brain functional recovery.^[^ ^26^ ^]^ Previous studies have shown that downregulation of hypoxia‐inducible factor‐1α (HIF‐1α) expression facilitates the polarization of macrophages and microglia from the proinflammatory M1 to the anti‐inflammatory M2 phenotype.^[^ ^27^ ^]^ In our study, we used RDPP and HIF‐1α staining to investigate the ability of MnO_2_‐Au‐mSiO_2_@Cur to alleviate hypoxia. CLSM images and fluorescence quantitative analysis demonstrated that the oxygen produced by catalysis significantly reduced intracellular hypoxia and downregulated the expression of HIF‐1α (**Figure** **6**A–C).

Cur, a natural compound with excellent anti‐inflammatory activity, can effectively promote the polarization of microglia from the M1 state to the M2 state.^[^ ^28^ ^]^ The anti‐inflammatory effect and ability of MnO_2_‐Au‐mSiO_2_@Cur to regulate microglial polarization were further investigated. Initially, BV2 cells were stimulated with H_2_O_2_ to induce a proinflammatory M1 state, while IL‐4 was used to stimulate BV2 cells to an anti‐inflammatory M2 state as a control (Figure S22, Supporting Information). CLSM images revealed that treatment with MnO_2_‐Au‐mSiO_2_@Cur resulted in increased M2 signaling and decreased M1 signaling in BV2 cells (Figure 6D,E). Immunofluorescence quantitative analysis revealed a significant increase in the M2/M1 ratio from 0.3 to 2.6 after treatment with MnO_2_‐Au‐mSiO_2_@Cur, indicating excellent microglial polarization from M1 to M2 induced by MnO_2_‐Au‐mSiO_2_@Cur (Figure 6F–H). Moreover, flow cytometry analysis confirmed the promotion of microglial polarization from M1 to M2 by MnO_2_‐Au‐mSiO_2_@Cur, which was consistent with the CLSM images (Figure 6I; Figure S23, Supporting Information). These results strongly demonstrated that the oxygen generated by catalysis and the anti‐inflammatory properties of MnO_2_‐Au‐mSiO_2_@Cur synergistically promoted the polarization of microglia from the M1 state to the M2 state.

### 2.7 Behavioral Evaluation of TBI Inflammation‐Targeted Therapy

**Figure 7:**
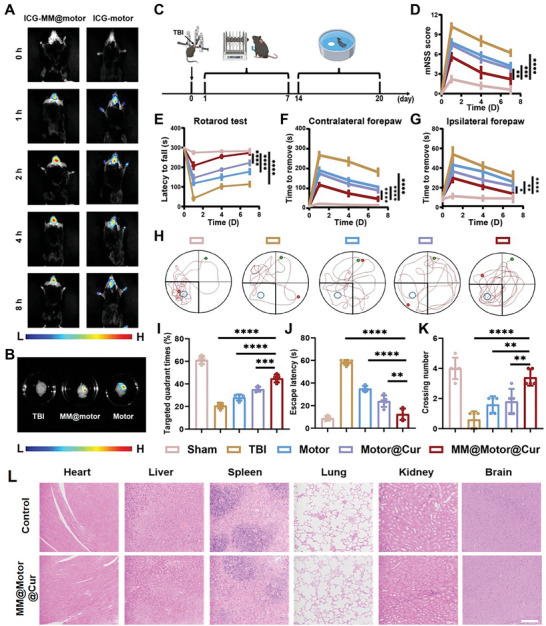
Active targeting of TBI‐related inflammatory tissue and behavioral improvement A) In vivo imaging system (IVIS) images showing TBI mice after injection of ICG‐labeled MM@motor and ICG‐labeled motor. B) IVIS images of brain tissue excised at 4 h postinjection with ICG‐labeled MM@motor and ICG‐labeled motor. C) Schematic showing the timeline of TBI mouse therapy. D) The mNSS was tested on days 1, 4, and 7 after the different treatments (*n* = 5). E) Rotor‐rod test results, F) latency to notice the contralateral forepaw, and G) latency to notice the ipsilateral forepaw of TBI mice were evaluated on days 1, 4, and 7 after different treatments (*n* = 5). H) Representative swimming trajectories of TBI mice after different treatments in the Morris water maze; I) quadrant, J) escape latency, and K) number of platform crossings (*n* = 5). L) H&E‐stained organ slices of the heart, liver, spleen, lung, kidney, and brain after different treatments on day 14. Scale bar, 200 µm. All the results are presented as the mean ± SD. Significant differences were assessed by one‐way ANOVA (**P < 0.01, ***P < 0.001, ****P < 0.0001).

TBI is known to trigger intense inflammatory responses, which can lead to cognitive decline and motor dysfunction.^[^ ^29^ ^]^ To address this issue, researchers have explored the potential of biomimetic MM‐modified MnO_2_‐Au‐mSiO_2_@Cur nanomotors to selectively accumulate in TBI regions due to their active targeting capability. Prior to intravenous injection into mice, hemolysis tests were conducted to assess the biocompatibility of MM@MnO_2_‐Au‐mSiO_2_@Cur, which demonstrated excellent biocompatibility (Figure S24, Supporting Information). Moreover, the stability of the MM@MnO_2_‐Au‐mSiO_2_@Cur nanomotor was further investigated. After one week of cultivation, the UV‒vis absorption of the nanoparticles did not significantly change (Figure S25, Supporting Information). DLS measurements showed that there was no noticeable change in nanoparticle size after incubation in different physiological microenvironments for seven days (Figure S26, Supporting Information). Additionally, after coculture with PBS, RPMI, or DMEM physiological culture media for seven days, the nanoparticles did not exhibit any significant aggregation (Figure S27, Supporting Information). Overall, these results collectively demonstrated the excellent stability of the MM@MnO_2_‐Au‐mSiO_2_@Cur nanomotors. Subsequently, researchers investigated the ability of MM@MnO_2_‐Au‐mSiO_2_@Cur to actively target brain injury tissue. Utilizing an in vivo imaging system (IVIS), the results revealed a greater presence of ICG fluorescence at the TBI sites when the cells were coated with biomimetic MM. These findings confirmed the ability of MM@MnO_2_‐Au‐mSiO_2_@Cur to actively target inflammation in TBI tissues (**Figure** **7**A,B). Moreover, brain sections from TBI mice after different treatments were obtained to further investigate the motion behaviors of the biomimetic nanomotors in the inflammatory microenvironment. The DiI‐labeled MM@Au‐MnO_2_‐mSiO_2_@Cur group exhibited greater penetration and better motion behaviors in the TBI microenvironment than did the DiI‐labeled MM@Au‐mSiO_2_@Cur group (Figure S28, Supporting Information), which further demonstrated the self‐propelled motion behaviors of the MM@Au‐MnO_2_‐mSiO_2_@Cur nanomotors in the pathological brain inflammation microenvironment.

Afterward, we conducted detailed behavioral studies on the recovery and treatment of TBI in mice using targeted nanomotors loaded with Cur (Figure 7C). To assess neurological function, we employed the modified neurological severity score (mNSS), which measures motor, sensory, balance, and reflex capabilities. The mNSS results indicated that the scores of the group treated with MM@MnO_2_‐Au‐mSiO_2_@Cur were significantly lower than those of the untreated TBI group, suggesting improved functional recovery in the treated mice (Figure 7D). To further evaluate motor and sensory function, we performed rotarod and adhesive tape removal tests. On day 1, the mice in the TBI group exhibited severe motor and balance deficits, as they quickly fell off the rotating rod. However, after 7 days of MM@MnO_2_‐Au‐mSiO_2_@Cur treatment, the mice exhibited similar performance to that of the sham group in the rotarod test, indicating significant recovery of motor and balance function (Figure 7E). Additionally, the time needed for the adhesive tape to be removed from the forepaws was measured. Compared to those in the sham group, the tape was removed from the mice in the TBI group, indicating impaired sensory function (Figure 7F,G). After treatment with MM@MnO_2_‐Au‐mSiO_2_@Cur, both paw removal times were reduced, suggesting apparent improvements in sensory and motor function following brain injury.

Subsequently, spatial learning and memory performance were assessed in mice after different treatments via Morris water maze tests^[^ ^30^ ^]^ (Figure 7H). Mice in the TBI group took more time to find the platform and even failed to locate it within the specified time after training. In contrast, the mice in the MM@MnO_2_‐Au‐mSiO_2_@Cur group spent less time searching for the platform and more time in the target quadrant than did the other treatment groups (Figure 7I,J). Moreover, as shown in Figure 7K, mice in the MM@MnO_2_‐Au‐mSiO_2_@Cur group crossed the platform position multiple times, indicating that MM@MnO_2_‐Au‐mSiO_2_@Cur promoted the recovery of spatial learning and memory abilities after TBI. Additionally, histopathological analysis via hematoxylin and eosin (H&E) staining revealed a reduction in lesion volume and restoration of damaged tissue in the TBI region following treatment with MM@MnO_2_‐Au‐mSiO_2_@Cur (Figure S29, Supporting Information). Moreover, we measured the brain water content of the TBI mice after different treatments (Figure S30, Supporting Information). The results showed that MM@MnO_2_‐Au‐mSiO_2_@Cur could significantly decrease the brain water content, which has an outstanding reparative effect on brain injury in TBI mice. Moreover, we conducted ICP‐MS detection to further investigate the biodistribution of the biomimetic nanomotors. The results revealed a rapid increase in the MM@MnO_2_‐Au‐mSiO_2_@Cur concentration in the brain at 6 h post‐injection, followed by a gradual decrease after 24 h (Figure S31, Supporting Information). This may suggest the metabolism of biomimetic nanomotors into the peripheral lymphatic system and bloodstream. The biomimetic nanomotors could potentially be transported out of the brain through typical cerebral metabolic pathways, such as the glymphatic system and cerebrospinal fluid (CSF) circulation system, and subsequently metabolized in major organs like the liver.^[^ ^19a^ ^]^ Overall, the metabolism of brain tissue is a complex system in which multiple pathways work together to ensure the normal metabolism and function of brain tissue.^[^ ^31^ ^]^ At 72 h post‐injection, the content of the biomimetic nanomotors in the brain significantly decreased, indicating that the majority of the nanomotors were transported out of the brain and metabolized by other organs and tissues.

Furthermore, the potential toxicity of MM@MnO_2_‐Au‐mSiO_2_@Cur was thoroughly investigated to assess its suitability for practical application. Histology, blood hematology, and blood biochemistry analyses were also conducted to assess the in vivo toxicity of the nanomedicine. Histology examination via H&E staining revealed no observable damage to major organs following treatment with MM@MnO_2_‐Au‐mSiO_2_@Cur, as depicted in Figure 7L. These findings indicate that the nanomedicine does not induce significant organ toxicity. To further investigate systemic biosafety after intravenous injection of MM@MnO_2_‐Au‐mSiO_2_@Cur, blood hematology analysis was performed. Blood hematology indicators, including red blood cell (RBC) count, platelet (PLT) count, white blood cell (WBC) count, hematocrit (HCT), neutrophil (Neu), hemoglobin (HGB), mean corpuscular hemoglobin (MCH), and mean corpuscular volume (MCV), did not significantly differ between the MM@MnO_2_‐Au‐mSiO_2_@Cur treatment group and the control group (Figure S32, Supporting Information). These findings suggested that MM@MnO_2_‐Au‐mSiO_2_@Cur had a minimal impact on systemic blood parameters, indicating good biocompatibility. Additionally, blood biochemistry analysis was conducted to measure aminotransferase (ALT), alanine aspartate aminotransferase (AST), blood urea nitrogen (BUN), and urea (UA) levels. Again, the results demonstrated that there were no distinct differences in blood biochemistry between the MM@MnO_2_‐Au‐mSiO_2_@Cur treatment group and the control group (Figure S33, Supporting Information). This finding suggested that the nanomedicine could not cause significant alterations in liver or kidney function, further supporting its excellent biosafety and biocompatibility in vivo. In conclusion, a comprehensive assessment of the toxicity of MM@MnO_2_‐Au‐mSiO2@Cur through histological examination, blood hematology analysis, and blood biochemistry demonstrated its excellent biosafety and biocompatibility when administered in vivo. Overall, these findings demonstrated that MM@MnO_2_‐Au‐mSiO_2_@Cur not only targets brain inflammation and enhances the recovery of damaged brain functions but also contributes to the potential clinical application of MM@MnO_2_‐Au‐mSiO_2_@Cur as a nanomedicine.

### 2.8 Regulation of ROS and Microglia/Macrophage Polarization In Vivo

**Figure 8:**
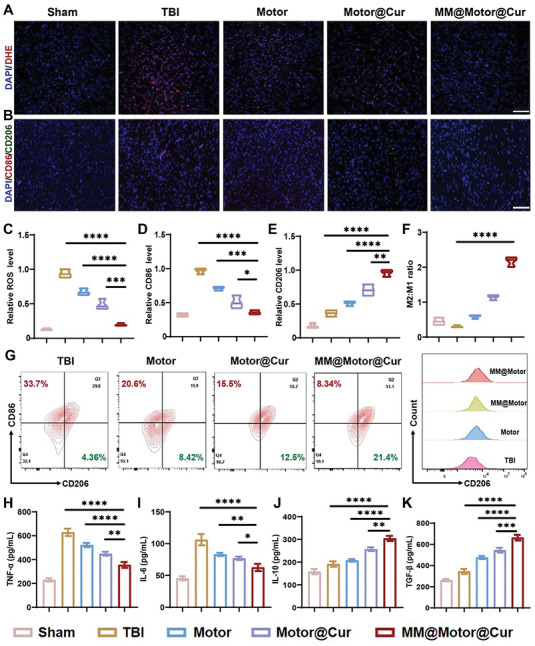
Regulation of ROS and microglial polarization from the proinflammatory M1 to the anti‐inflammatory M2 phenotype in TBI mice. A) DHE staining of mice after different treatments. Scale bar, 100 µm. B) Immunofluorescence staining of CD206 (M2 marker) and CD86 (M1 marker) in the brain after different treatments. Quantification of C) ROS levels, D) CD86 levels, E) CD206 levels, and F) M2/M1 microglial ratios. G) FCM analysis of microglial polarization from M1 to M2 after different treatments. Cytokine levels in mouse sera, including H) TNF‐α, I) IL‐6, J) IL‐10, and K) TGF‐β, were detected by ELISA kits after different treatments. All the results are presented as the means ± SDs (*n* = 3). Significant differences were assessed by one‐way ANOVA (*P < 0.05, **P < 0.01, ***P < 0.001, ****P < 0.0001).

TBI can induce a strong and harmful inflammatory stress response in brain tissues characterized by elevated production of ROS and the polarization of macrophages and microglia toward a proinflammatory state.^[^ ^32^ ^]^ Building upon successful intracellular ROS clearance experiments, we further investigated the ability of MM@MnO_2_‐Au‐mSiO_2_@Cur to reduce ROS levels in TBI‐affected brain regions. As shown in **Figure** **8**A, a remarkable decrease in ROS levels was detected in the brain tissue following treatment with MM@MnO_2_‐Au‐mSiO_2_@Cur compared to that in the TBI group, highlighting the strong ROS scavenging capacity of MM@MnO_2_‐Au‐mSiO_2_@Cur in the context of TBI. This reduction in ROS was further confirmed through quantitative analysis (Figure 8C). To investigate the M2/M1 ratio in TBI tissues after different treatments, we performed CD206/CD86 immunofluorescence co‐staining (Figure 8B). As shown in Figure 8D–F, the proportion of microglia/macrophages exhibiting an M1 phenotype significantly increased in the TBI group, while the proportion of microglia/macrophages exhibiting an M2 phenotype substantially decreased. However, after treatment with MM@MnO_2_‐Au‐mSiO_2_@Cur, the M2/M1 ratio of microglia/macrophages was notably elevated, indicating a dampened proinflammatory response and an enhanced anti‐inflammatory response in the TBI region. Quantification of the CD206^+^CD86^−^ (M2) and CD206^−^CD86^+^ (M1) ratios in TBI tissues via FCM analysis yielded similar trends to those shown in the CLSM images presented in Figure 8G and Figure S34 (Supporting Information). Furthermore, we examined the levels of anti‐inflammatory factors (TGF‐β and IL‐10) and proinflammatory factors (IL‐6 and TNF‐α) to investigate whether MM@MnO_2_‐Au‐mSiO_2_@Cur could modulate the release of inflammation‐related molecules. As depicted in Figure 8H–K, the ELISA results demonstrated an increase in the IL‐10 and TGF‐β levels and a decrease in the IL‐6 and TNF‐α levels, indicating the robust enhancement of the anti‐inflammatory effect of MM@MnO_2_‐Au‐mSiO_2_@Cur in the treatment of TBI. Collectively, these in vivo findings demonstrated that MM@MnO_2_‐Au‐mSiO_2_@Cur nanoparticles exhibit efficient ROS scavenging capacity, promote macrophage or microglial polarization from the M1 to the M2 phenotype, enhance the anti‐inflammatory response, and facilitate the repair of damaged brain tissue.

## 3 Conclusion

In summary, we developed a novel biomimetic self‐propelled nanomotor with an octopus‐type asymmetric structure for cascade‐targeted anti‐inflammatory therapy for central nervous system inflammatory diseases. Through the fabrication of an MM coating, these biomimetic nanomotors effectively accumulate in regions of brain inflammation, crossing the BBB to reach their target sites. In BBB organoid model experiments, we observed that the biomimetic‐modified nanomotors were capable of traversing the BBB and preferentially localized to sites of inflammation. The biomimetic self‐propelled nanomotors exhibited high CAT and SOD enzymatic activities, enabling the effective removal of overexpressed ROS. Simultaneously, the nanomotors generated a large number of oxygen bubbles as a self‐driving force, facilitating deep penetration into the brain tissue. Additionally, the encapsulated Cur in the nanomotors demonstrated enhanced anti‐inflammatory properties and promoted the polarization of microglia. Experimental data obtained by our research team demonstrated that MM@MnO_2_‐Au‐mSiO_2_@Cur nanomaterials successfully targeted brain inflammation, leading to a reduction in ROS levels and promoting the polarization of macrophages and microglia from the proinflammatory M1 phenotype to the anti‐inflammatory M2 phenotype in the TBI region. Importantly, animal experiments showed that TBI mice treated with the MM@MnO_2_‐Au‐mSiO_2_@Cur nanomotor exhibited restoration of behavioral deficits and spatial learning ability and improved cognitive function. Furthermore, the biomimetic self‐propelled nanomotors demonstrated excellent biosafety both in vitro and in vivo, indicating their potential for practical application and clinical translation. Overall, our study highlights the effectiveness and safety of the developed biomimetic self‐propelled nanomotors for targeted anti‐inflammatory therapy in central nervous system inflammatory diseases, providing a promising approach for future therapeutic interventions.

## 4 Experimental Section

### Synthesis of MnO_2_‐Au‐mSiO_2_@Cur Janus NPs (JNs)

In the experimental procedure, 1 mL of the as‐prepared Au‐mSiO_2_ JNs was combined with 4 mL of 1% polyvinylpyrrolidone (PVP) solution. The solution was stirred gently for 15 min at room temperature. Subsequently, 1.5 mL of KMnO_4_ solution (4 mg mL^−1^) was added to the solution, and the mixture was allowed to react for 2 h. The resulting MnO_2_‐Au‐mSiO_2_ nanomaterials were generated by centrifugation and further washed twice with water. To load Cur into the nanoparticles, a solution of Cur (5 mg mL^−1^) was added to the MnO_2_‐Au‐mSiO_2_ nanoparticles in ethanol. The mixture was stirred gently overnight. Finally, the MnO_2_‐Au‐mSiO_2_@Cur nanoparticles were obtained by centrifugation and washed with ethanol and water to remove any excess free Cur.

### Synthesis of MM@MnO_2_‐Au‐mSiO_2_@Cur NPs

First, the MM samples were dispersed in water and passed through 400 nm and then 200 nm long polycarbonate porous membranes using an Avanti mini extruder. This extrusion process helped to obtain MM vesicles with a specific size distribution. Subsequently, the MnO_2_‐Au‐mSiO_2_@Cur nanomotors were mixed with the MM vesicles obtained in the previous step. The mixture was then subjected to further extrusion through a 200 nm polycarbonate porous membrane using an Avanti mini extruder. This extrusion process was performed 21 times to ensure efficient incorporation and encapsulation of the MnO_2_‐Au‐mSiO_2_@Cur nanomotors within the MM vesicles. As a result, the final product obtained was MM@MnO_2_‐Au‐mSiO_2_@Cur, where the MM vesicles contained the incorporated MnO_2_‐Au‐mSiO_2_@Cur nanomotor.

### Animal Experiments

Male C57BL/6J mice (8–10 weeks old, 20–22 g) were purchased from Beijing Vital River Laboratory Animal Technology Co., Ltd. All animal studies were conducted according to the National Institute Guide for the Care and Use of Laboratory Animals and the Animal Ethics Committee of Tianjin University (approval number: TJUE‐2022‐210).

### TBI Model Establishment and Treatment

TBI mouse models were established using an electromagnetically driven controlled cortical impact (CCI) device guided by a brain stereotactic apparatus (Beijing Zhongshi Dichuang Technology Development Co., Ltd.). First, the animals were intraperitoneally anesthetized with 1% pentobarbital sodium. The bregma and the right parietal bone of the mice were then exposed. A 2 mm burr hole was made 2 mm posterior to the bregma and 2 mm to the right of the midline using a cranial drill. The CCI impact procedure was performed using a 2 mm diameter flat impact tip, with an impact velocity of 4 m s^−1^ and an impact depth of 2 mm. The mice in the sham group underwent the same procedures as those described above but without the impact procedure. For treatment, TBI mice were injected with PBS, MnO_2_‐Au‐mSiO_2_ (Motor), MnO_2_‐Au‐mSiO_2_@Cur (Motor@Cur), or MM@MnO_2_‐Au‐mSiO_2_@Cur (MM@Motor@Cur) (100 µL, 1 mg mL^−1^) via the tail vein. Behavioral capacity was evaluated on days 1, 4, and 7 using the modified neurological severity score (mNSS), rotor‐rod analysis, and adhesive tape removal tests. On day 21, the mice in each group were sacrificed, and brain tissues were removed for assessment of brain tissue recovery, ROS levels, and microglial polarization. This was performed through H&E staining, DHE staining, and CD86 (Novus, NBP2‐25208)/CD206 (Abcam, ab300621) immunofluorescence staining, followed by FCM analysis. In addition, the levels of TGF‐β, IL‐10, IL‐6, and TNF‐α in mice after different treatments were measured using ELISA kits.

## Conflict of Interest

The authors declare no conflict of interest.

## Supporting information

Supporting Information

## Data Availability

The data that support the findings of this study are available in the supplementary material of this article.
