# Supplementary material for: Biomimetic Self‐Propelled Asymmetric Nanomotors for Cascade‐Targeted Treatment of Neurological Inflammation
Source: Adv Sci (Weinh). 2024 Mar 9;11(22):2310211. doi: 10.1002/advs.202310211 (PMC11165487; doi:10.1002/advs.202310211)
Supplement: Supplementary file 1 — Supporting Information [file ADVS-11-2310211-s001.pdf]

ADVANCED SCIENCE
Open Access

Supporting Information

for *Adv. Sci.*, DOI 10.1002/advs.202310211

Biomimetic Self-Propelled Asymmetric Nanomotors for Cascade-Targeted Treatment of Neurological Inflammation

Jiamin Ye, Yueyue Fan, Yaoguang She, Jiacheng Shi, Yiwen Yang, Xue Yuan, Ruiyan Li, Jingwen Han, Luntao Liu, Yong Kang* and Xiaoyuan Ji**

Supporting Information

Biomimetic Self-Propelled Asymmetric Nanomotors for Cascade-Targeted Treatment of Neurological Inflammation

Jiamin Ye, Yueyue Fan, Yaoguang She, Jiacheng Shi, Yiwen Yang, Xue Yuan, Ruiyan Li, Jingwen Han, Luntao Liu, Yong Kang*, Xiaoyuan Ji**

J. Ye, Y. Fan, Y. Kang, J. Shi, Y. Yang, X. Yuan, R. Li, J. Han, X. Ji
Academy of Medical Engineering and Translational Medicine, Medical College, Tianjin University, Tianjin 300072, China.
E-mail: liuluntao@irm-cams.ac.cn (L. L.); kangyong@tju.edu.cn (Y.K.); jixiaoyuan@tju.edu.cn (X.J.)

Y. She
Department of General Surgery, the First Medical Center, Chinese People's Liberation Army General Hospital, Beijing 100853, China

L. Liu
Tianjin Key Laboratory of Radiation Medicine and Molecular Nuclear Medicine, Institute of Radiation Medicine, Chinese Academy of Medical Sciences and Peking Union Medical College, Tianjin, China

X. Ji
Medical College, Linyi University, Linyi 276000, China

Experimental Section

Materials. $HAuCl_4 \cdot 3H_2O$ (99.9%), sodium citrate (99%), polyvinylpyrrolidone (PVP, Mw = 10,000), tetraethyl orthosilicate (TEOS, 99.999%), 4-mercaptophenylacetic acid (4-MPAA, 97%), $[Ru(dpp)_3]Cl_2$ (RDPP), and potassium permanganate ($KMnO_4$, 99%) were purchased from Sigma–Aldrich. Polyacrylic acid (PAA, Mw = 5,000) was purchased from J&K Chemical Ltd. Sodium hydroxide (NaOH), ammonium hydroxide ($NH_3 \cdot H_2O$), 3,3′,5,5′-tetramethylbenzidine (TMB), hydrogen peroxide (H_2O_2), curcumin (Cur), ethanol (EtOH), and isopropanol (IPA) were purchased from Aladdin. A Cell Counting Kit-8 (CCK-8) was obtained from MedChemExpress (MCE, USA). The catalase (CAT) assay kit, total superoxide dismutase (SOD) assay kit, 2’,7’-dichlorofluorescin diacetate (DCFH-DA), annexin V-FITC/PI apoptosis detection kit, LysoTracker Red, and Hoechst 33342 were obtained from Beyotime Biotechnology. A JC-1 mitochondrial membrane potential kit was purchased from AbMole. The ultrapure water (18.2 MΩ cm) used in all the experiments was obtained from a Milli-Q water purification system (Millipore Co., USA). All the chemical reagents listed above were used directly in the experiments without further purification.

Synthesis of Au NPs. AuNPs **(**50 nm) were designed *from* 17 nm AuNPs through a seed-mediated growth method. First, 17 nm Au nanoparticles were synthesized by the citrate reduction method. Afterward, 0.5 mL of $HAuCl_4$ (10 mg/mL) was dissolved in 100 mL of water and heated to 150°C under vigorous stirring for 10 min. Finally, the as-prepared 13 nm AuNPs (4 mL) and sodium citrate dihydrate (0.4 mL, 1% w/w) were added to the mixture and reacted for another 15 min to further obtain 50 nm AuNPs.

Synthesis of Au-mSiO$_2$ JNs. The as-prepared 20 mL of AuNPs was centrifuged (4600 rpm, 10 min) and concentrated to 5 mL. Afterward, 5 mL of concentrated AuNP solution was transferred to 25 mL of IPA under vigorous stirring, after which 4-MPAA (200 μL, 0.645 mM in water) and PAA (720 μL, 5 mM in ethanol) were added to the mixture. After 45 minutes of reaction, 9 mL of TEOS (8.9 mM) and 0.9 mL of $NH_3 \cdot H_2O$ were introduced into the system and allowed to react for another 4 h under stirring. The Au-SiO_2 JNs were collected by centrifugation (6000 rpm, 12 min) and washed with IPA and water. Afterward, the PVP solution (1 mL, 4 wt%) was added to the as-prepared Au-SiO_2 JNs, which were heated to 100°C for 1 h to obtain Au-mSiO_2 JNs.

Isolation of the macrophage membrane (MM). RAW 264.7 cells were cultured in high-glucose DMEM supplemented with 1% penicillin–streptomycin and 10% fetal bovine serum (FBS). The cells were incubated in a humidified incubator at 37°C with 5% CO_2. To prepare the macrophage lysate, RAW 264.7 cells (1×10^8) were harvested and resuspended in 2 mL of hypotonic lysing buffer. The macrophages were then subjected to repeated freeze–thaw cycles to further lyse and release the cellular contents. The precipitated intracellular components of the lysed macrophage suspension were removed by centrifugation at 800 rpm for 20 min. The resulting supernatant, which contained the desired extracellular fraction, was collected. To remove organelles and other cellular debris, the supernatant was centrifuged at 10,000 rpm. Finally, the supernatant was subjected to high-speed centrifugation at 100,000 rpm for 30 min at 4°C to collect the MM samples. These MM samples were carefully preserved at -80°C for further analysis and experimentation.

Characterization of MM@MnO_2-Au-mSiO_2@Cur. The morphology of the materials was assessed via transmission electron microscopy (TEM) following negative staining with a 1% (w/v) phosphotungstic acid solution. The zeta potential and hydrodynamic size distribution were detected using dynamic light scattering (DLS) measurements. X-ray diffraction and X-ray photoelectron spectroscopy were applied to determine the composition and chemical structure of the materials, respectively. The presence of typical markers, such as CD11b (Abcam, ab133357) and CD44 (Abcam, ab189524), in MM@MnO_2-Au-mSiO_2@Cur was confirmed through western blotting analysis. The oxygen generation capacity was measured using a portable dissolved oxygen meter, with dissolved oxygen levels recorded over a period of 900 seconds.

CAT and SOD enzyme activity assays. The CAT- and SOD-mimicking enzyme activities of MnO_2-Au-mSiO_2 and MnO_2-Au-mSiO_2@Cur were determined by CAT and SOD kits (Beyotime, China) according to the manufacturer's instructions. For H_2O_2 scavenging, different concentrations of MnO_2-Au-mSiO_2@Cur were mixed with a 1 mM H_2O_2 solution. The mixture was allowed to stand at room temperature for 10 minutes before being centrifuged (4600 rpm, 10 min) to obtain the supernatant. The TMB solution and $FeCl_3\cdot4H_2O$ were subsequently added to the supernatant and allowed to react for another 10 minutes. A digital camera was used to capture the color of the TMB solution, and the UV–vis spectra were examined to determine H_2O_2 consumption. Furthermore, the stopping solution technique was carried out in the same way as the previous method, except that the stopping solution (10% H_2SO_4) was added to the

above solution immediately after the 10 min reaction with H_2O_2. Moreover, electron spin resonance (ESR) measurements were performed in which 5,5-dimethyl-1-pyrroline N-oxide (DMPO) was used as the spin-trapping agent.

Nanomotor motion behavior. The motion behavior of the nanomotors was measured using a dark field microscope (Nikon, Japan) and analyzed with CUDA Spot Tracker software. The corresponding mean square displacement (MSD) of the nanomotors was calculated according to the following equation: $MSD = (x(\Delta t)-x(0))^2 + (y(\Delta t))-y(0))^2$. The diffusion coefficient (D_{eff}) was obtained through the Stokes–Einstein equation, $D = MSD/(i \times \Delta t)$, where Δt represents the time interval and *i* is the dimensional index. In this case, *i* is equal to 4 for two-dimensional analysis.

Intracellular motion study of the nanomotors. To assess the ability of MnO_2-Au-$mSiO_2$@Cur to escape lysosomes, a lysosome escape capacity assay was performed in vitro. Specifically, SH-SY5Y cells (1×10^5 cells/mL) were seeded in confocal dishes and allowed to adhere for 24 h. FITC-labeled MnO_2-Au-$mSiO_2$@Cur nanoparticles were subsequently added to the cell dishes and incubated for 1 h, with or without prior treatment with H_2O_2. Subsequently, the cells were stained with LysoTracker (red fluorescence) and incubated for 30 minutes at 37°C, followed by three washes with PBS. The cell nuclei were subsequently stained with DAPI and incubated for an additional 15 minutes, followed by three washes with PBS. Finally, representative cell images were captured using CLSM.

To evaluate the motion of the nanomotors, SH-SY5Y cell-based 3D cellular spheroids were established. Initially, 50 µL of 1% agarose was added to each well of a 96-well plate and exposed to UV light for at least 30 minutes before use. Then, 200 µl of the SH-SY5Y cell suspension (1×10^3 cells/mL) was seeded into each well of a 96-well plate. After incubating for 10 days, the SH-SY5Y cell-based 3D cellular spheroids were successfully formed and selected for further permeation behavior testing. Specifically, FITC-labeled nanomotors were added to each well, followed by the introduction of 1 mM H_2O_2 to induce oxidative stress. After incubating for 6 h, the 3D cellular spheroids were removed from the wells and washed three times with PBS. The 3D cellular spheroids after different treatments were then observed using CLSM. As a control, FITC-labeled Au-$mSiO_2$ was also added in the same way as the FITC-labeled nanomotors, and the samples were subsequently observed via CLSM.

In vitro cell cytotoxicity Human neuroblastoma (SH-SY5Y) and mouse microglia (BV2) were cultured in high-glucose DMEM supplemented with fetal bovine serum (FBS, 10%) and 1% penicillin streptomycin at 37°C and 5% CO_2. The cell cytotoxicity of the MM@MnO_2-Au-mSiO_2@Cur nanomotors was assessed using a cell counting kit-8 (CCK-8) kit. Initially, the SH-SY5Y and BV2 cells were seeded into 96-well plates at a density of 5×10^4 cells/well and incubated for 24 h. Subsequently, the cells were exposed to varying concentrations of MM@MnO_2-Au-mSiO_2@Cur for another 24 h. Then, 10 μL of CCK-8 solution was added to each well, and the cells were incubated for 1 h. The absorption (at 450 nm) in each well was detected by a microplate reader. Moreover, to evaluate the therapeutic effect of different concentrations of MM@MnO_2-Au-mSiO_2@Cur on H_2O_2-induced oxidative stress, a CCK-8 test was performed to evaluate cell viability. The experimental procedure was similar to the above methods except that the cells were pretreated with 1 mM H_2O_2 for 1 h.

3D BBB organoid model. BBB organoid models were constructed by multimaterial printing using digital light processing (DLP). After maintaining a slice thickness of 100 μm, a light intensity of 10 mW/cm^2, and an exposure time of 10 s, cell-loaded GelMA bioink (pericytes, astrocytes) was added to the DLP printer feeder tank, and after printing for 20 layers, the mixture was replaced with cell-loaded AlgMA bioink (vascular endothelial cells + neural progenitors). The GelMA substrate was used to construct a duct structure with a diameter of 400 μm, and then, 20 layers were printed with cell-loaded GelAM bioink to obtain the final BBB early developmental model. After coincubation for 14 days, CD31 was selected as an endothelial cell marker, GFAP as an astrocyte marker, and NG2 as a peripheral cell marker. Immunofluorescence staining was performed on the BBB, and the distribution of various cells was observed using confocal laser scanning microscopy (CLSM) to characterize the morphology of the cells. Moreover, for the junction structure of BBB organoid models, CD31 was selected as the endothelial cell marker, ZO-1 was selected as the marker for BBB-specific junction protein, and laminin was selected as the marker for BBB basal protein. After immunofluorescence staining, the tight junction structure and basal structure of the BBB organoid models were observed via CLSM.

Cellular Uptake. SH-SY5Y cells (1×10^5 cells/mL) were seeded and cultured in confocal dishes for 24 h. The cells were then treated with FITC- and DiI-labeled MM@MnO_2-Au-mSiO_2@Cur both in the absence and presence of additional H_2O_2 for 4 h. Afterwards, the nuclei

of the SH-SY5Y cells were stained with DAPI for 15 minutes, and cellular images were acquired using CLSM.

Transwell transportation assay To investigate the penetration of MnO_2-Au-mSiO_2@Cur and MM@MnO_2-Au-mSiO_2@Cur, cocultures of SH-SY5Y and bEnd.3 cells were performed in 24-well Transwell chambers (polycarbonate filter, Corning, USA), in which bEnd.3 cells were inoculated into the upper layer and SH-SY5Y cells were inoculated into the lower layer of the Transwell chamber. A transendothelial electrical resistance (TEER) greater than 200 $\Omega.cm^2$ indicated successful construction of the in vitro BBB model. Then, FITC-labeled MnO_2-Au-mSiO_2@Cur and FITC-labeled MM@MnO_2-Au-mSiO_2@Cur solutions were added to the upper chamber with or without additional 1 mM H_2O_2 and cultured for 4 h. After 4 h of incubation, an in vivo imaging system (IVIS) was used to detect the fluorescent signals in the lower chambers. Moreover, the Mn concentration in the lower chamber after the different treatments was measured via ICP–MS.

Intracellular ROS scavenging capacity. To investigate the ROS scavenging capacity of the strains, SH-SY5Y cells (1×10^5 cells/mL) and BV2 cells (1×10^5 cells/mL) were separately seeded into a 6-well plate and incubated at 37°C for 24 h. Subsequently, the cells were precultured with 1 mM H_2O_2 for 1 h, followed by another 6 h incubation with PBS, Au-mSiO_2, MnO_2-Au-mSiO_2, or MnO_2-Au-mSiO_2@Cur. After the medium was removed and the cells were washed with PBS, the intracellular ROS levels were determined using 2',7'-dichlorofluorescein diacetate (DCFH-DA) kits (Beyotime) after different treatments. Additionally, the quantitative intracellular ROS level was measured using flow cytometry (FCM) analysis.

Intracellular mitochondrial membrane potential detection. A 5,5',6,6'-tetrachloro-1,1',3,3'-tetraethylbenzimidazolylcarbocyanine iodide (JC-1) kit was used to evaluate changes in the intracellular mitochondrial membrane potential. Specifically, SH-SY5Y cells (1×10^5 cells/mL) were seeded into confocal dishes and incubated for 24 h. Subsequently, the cells were treated with 1 mM H_2O_2 for 1 h and then incubated with PBS, Au-SiO_2, MnO_2-Au-mSiO_2, or MnO_2-Au-mSiO_2@Cur (100 µg/mL) for an additional 6 h. The cells were then washed three times with PBS and stained with JC-1 for 20 minutes. Finally, after different treatments, the SH-SY5Y cells were visualized using CLSM.

Apoptosis analysis SH-SY5Y cells (1×10^6 cells/mL) were seeded into a 6-well plate and incubated for 24 h. The cells were then treated with 1 mM H_2O_2. Subsequently, the SH-SY5Y cells in each well of the 6-well plates were mixed with PBS, Au-mSiO$_2$, MnO$_2$-Au-mSiO$_2$, or MnO$_2$-Au-mSiO$_2$@Cur (100 µg/mL). Finally, an Annexin V-FITC/PI kit (Beyotime) and flow cytometry (FCM) analysis were used to evaluate cell apoptosis after the different treatments.

Intracellular O_2 generation. The intracellular O_2 generation capacity was measured using an RDPP probe. SH-SY5Y cells (1×10^5 cells/mL) were seeded into confocal dishes and incubated at 37°C and 5% CO_2 for 12 h. Next, the SH-SY5Y cells were incubated with PBS, Au-mSiO$_2$, MnO$_2$-Au-mSiO$_2$, or MnO$_2$-Au-mSiO$_2$@Cur (100 µg/mL) in a hypoxic incubator for 6 h, followed by the addition of 1 mM H_2O_2. After incubating for another 6 h, the SH-SY5Y cells were treated with RDPP (1 µM) for 20 minutes and then washed three times with PBS. Finally, fluorescence images of the SH-SY5Y cells after different treatments were obtained using CLSM.

In vivo imaging ICG-labeled Motor and ICG-labeled MM@Motor@Cur (100 µL, 1 mg/mL) were injected into TBI mice via the tail vein. The TBI mice were then monitored using the IVIS system at 0, 1, 2, 4, and 8 h after injection. Furthermore, the mice were euthanized to extract brain tissue, and the distribution of ICG-labeled samples in the brain was visualized using an IVIS system.

Morris water maze (MWM) test The MWM test was also conducted to evaluate the recovery of memory and spatial learning in TBI mice following various treatments. Each mouse underwent a 5-day training period, with four trials per day, to locate the hidden platform. If a mouse failed to find the platform within 60 seconds, it was gently guided to the platform and allowed to stay there for 10 seconds to facilitate memory formation. After the 5-day training period, the platform was removed, and the mice from each group were allowed to swim freely for 60 seconds. The duration of time spent in the target quadrant, the escape latency, and the number of times the mice crossed over the previous platform location were measured and recorded as indicators of spatial learning and memory capacity.

Brain water content: The damaged side of the mouse brain was collected after different treatments, weighed (W_1) and then dried for 3 days. The dried brain tissue was weighed as W_2,

and the brain water content was determined by the following equation: Brain water content (%) = $(W_1 - W_2)/W_1 \times 100\%$.

Biodistribution analysis The TBI mice were intravenously injected with MM@MnO_2-Au-mSiO_2@Cur (5 mg/kg). The organs, including the heart, liver, spleen, lung, kidney, and brain, were collected at 6 h, 24 h, 48 h, and 72 h after injection and then analyzed via ICP–MS (n = 3).

Statistical Information. All the data were compared between groups using GraphPad Prism (version 8.2.1). The experimental results are presented as the means ± standard deviations (SD). A statistically significant difference was determined by using one-way ANOVA. The data were considered statistically significant when the *P* value was less than 0.05. The asterisk (*) denotes the level of significance, with * representing P <0.05, ** representing $P < 0.01$, *** representing $P < 0.001$, and **** representing $P < 0.0001$.

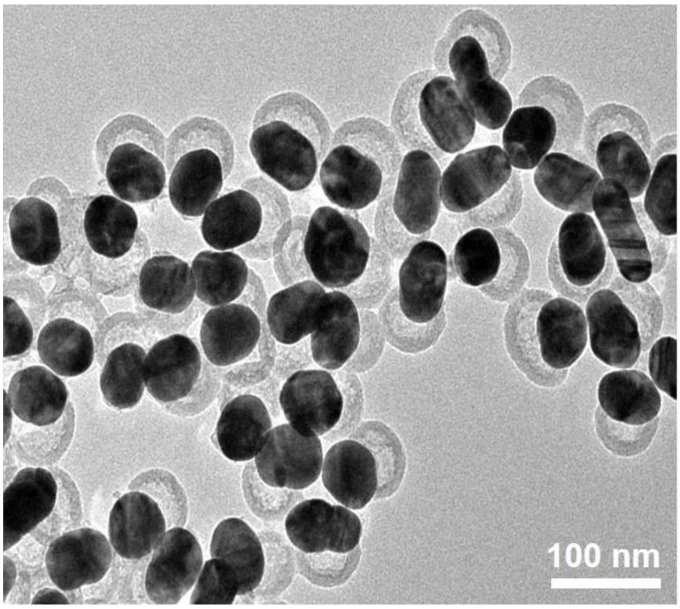

Figure S1. TEM image of Au-mSiO_2.

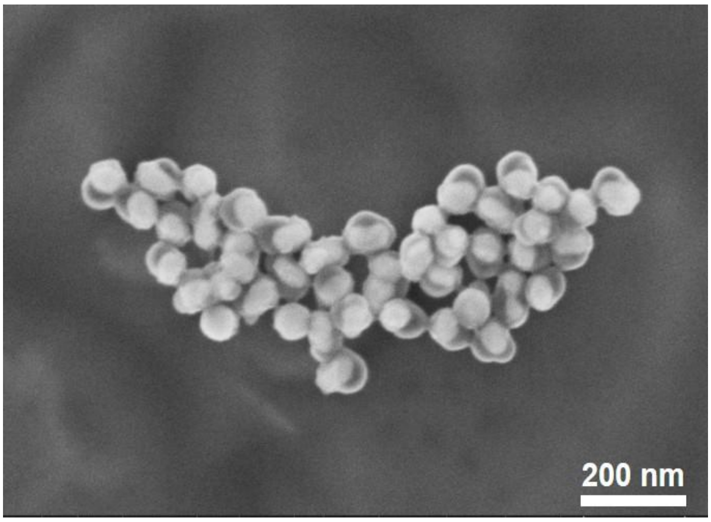

Figure S2. SEM image of MnO_2-Au-mSiO_2.

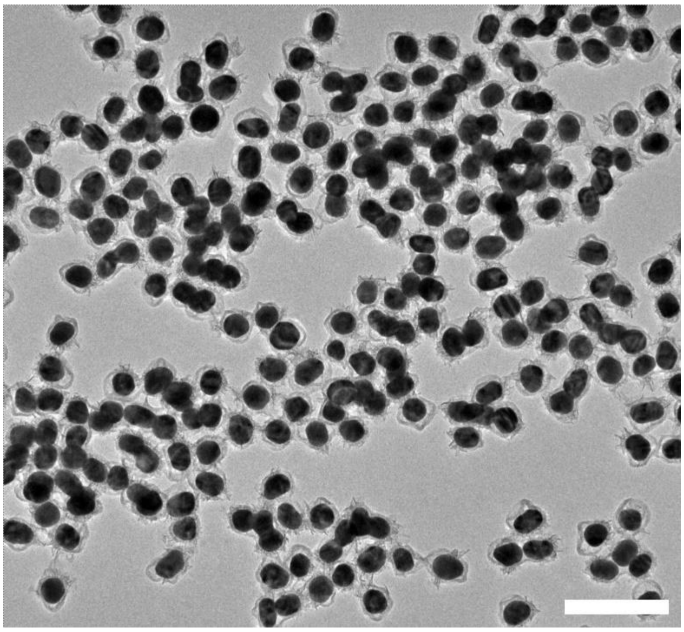

Figure S3. Low magnification TEM images of MnO_2-Au-mSiO_2. Scale bar = 200 nm.

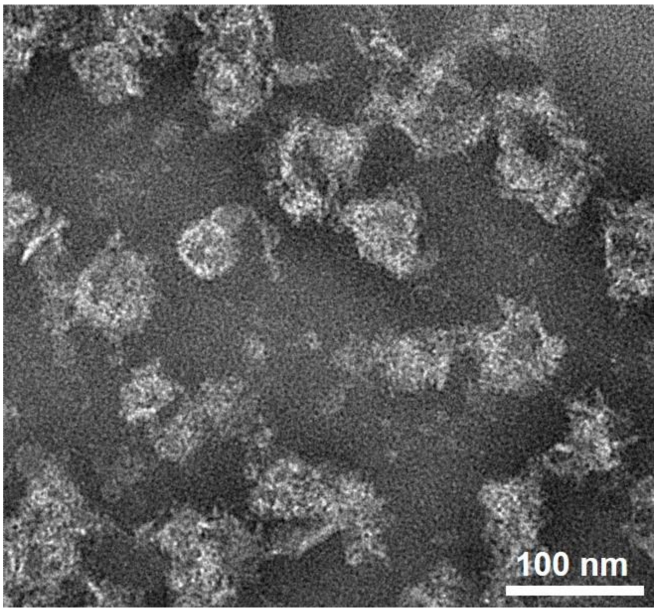

Figure S4. TEM image of MM vesicles.

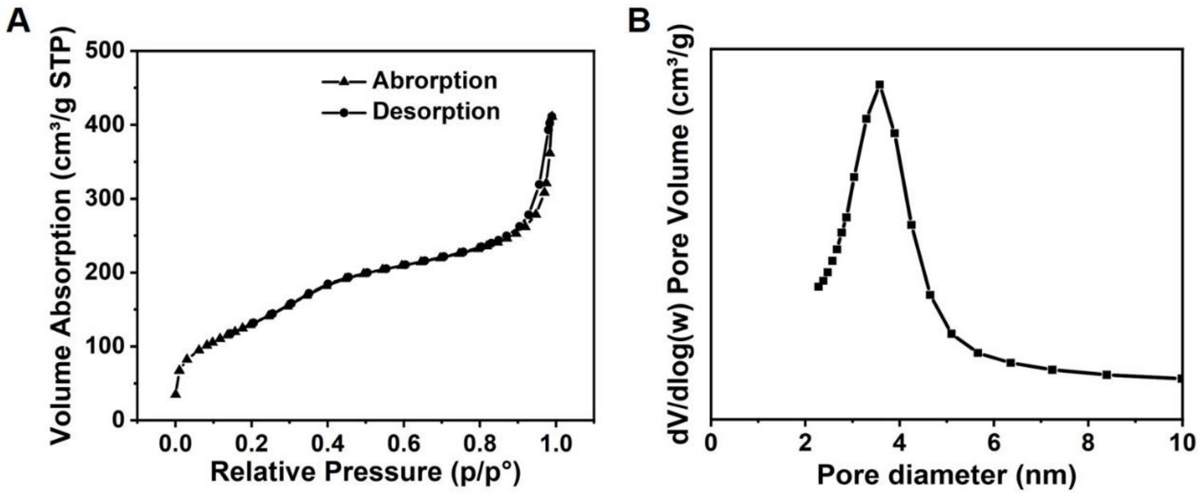

Figure S5. (A) Volume absorption and desorption of MnO_2-Au-mSiO_2. (B) Pole diameter analysis of MnO_2-Au-mSiO_2.

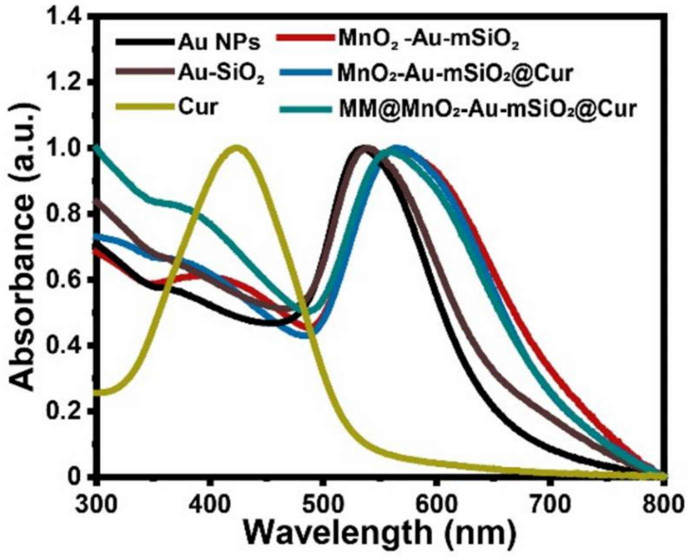

Figure S6. UV−vis spectra of different sample.

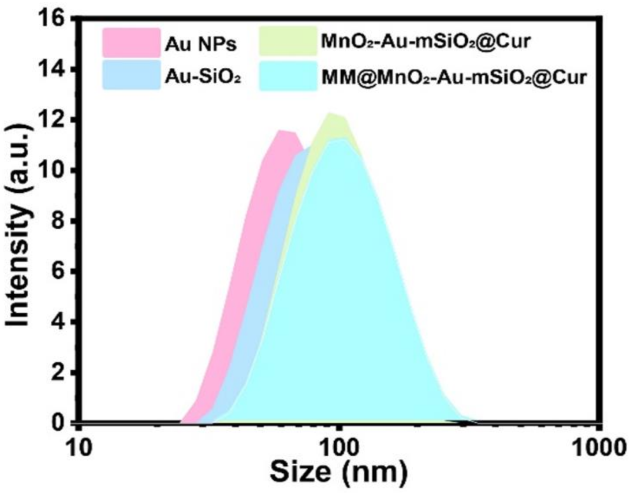

Figure S7. DLS analysis of different samples.

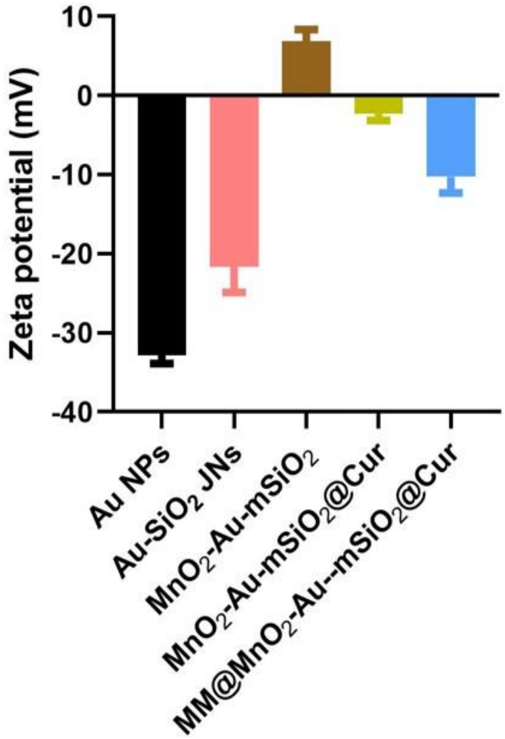

Figure S8. Zeta potential of different samples.

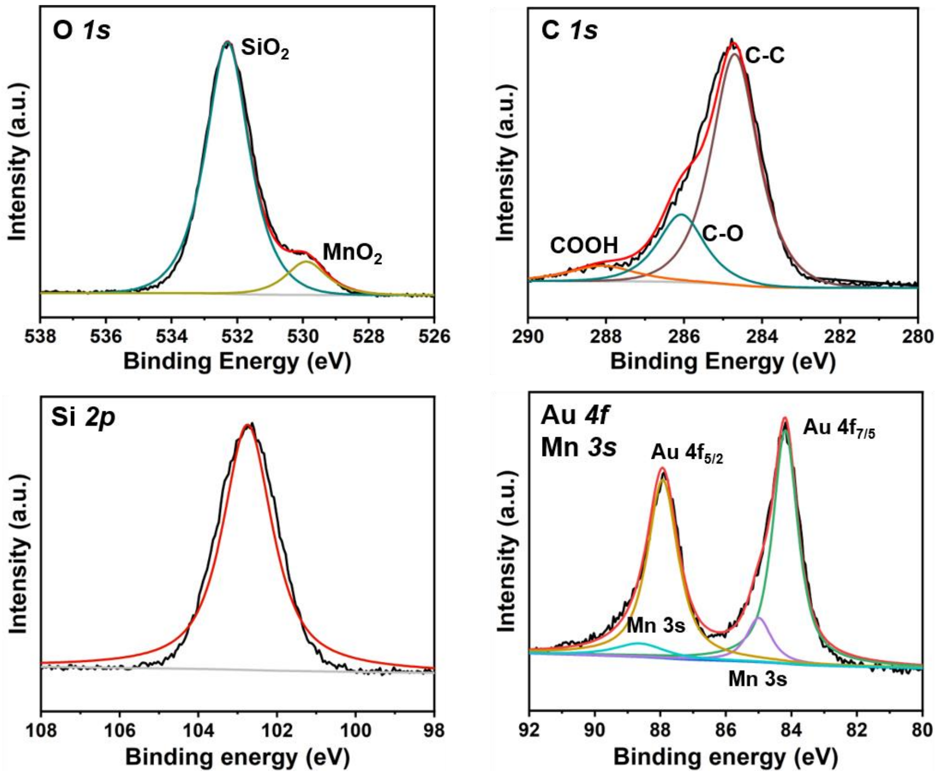

Figure S9. High-resolution XPS spectrum of O 1 s, C 1 s, Si 2p, Au 4f and Mn 3 s.

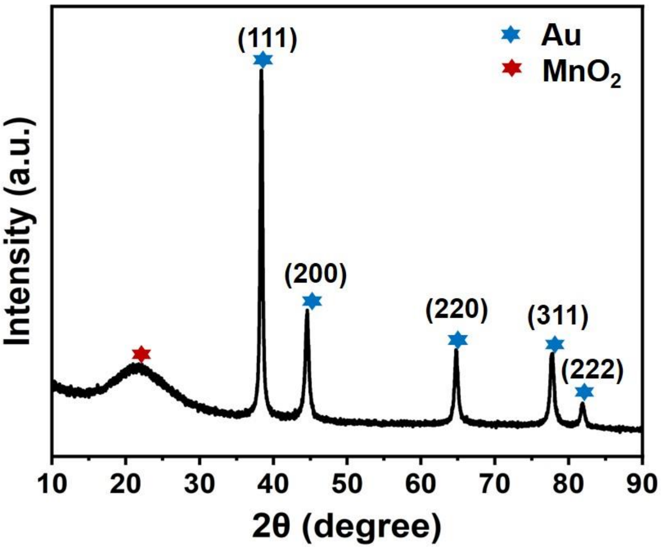

Figure S10. XRD spectrum of MnO_2-Au-mSiO_2.

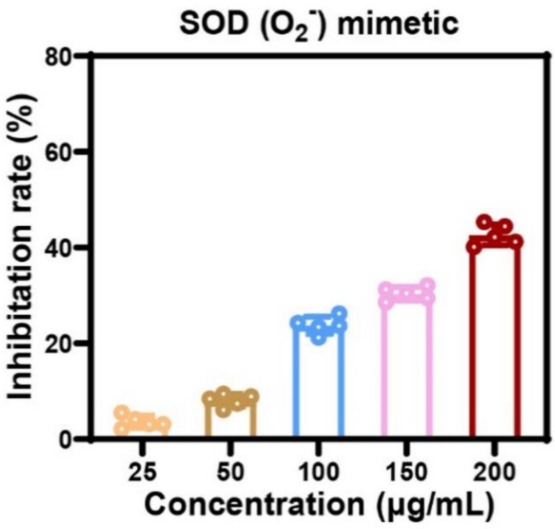

Figure S11. SOD mimetic activity of different concentrations of MnO_2-Au-mSiO_2 (n = 5).

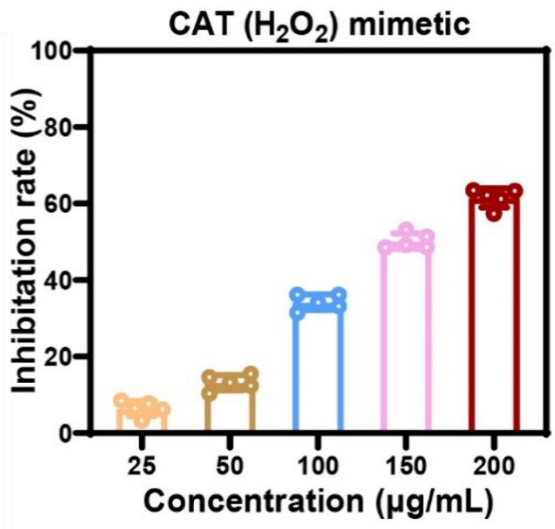

Figure S12. CAT mimetic activity of different concentrations of MnO_2-Au-mSiO_2 (n = 5).

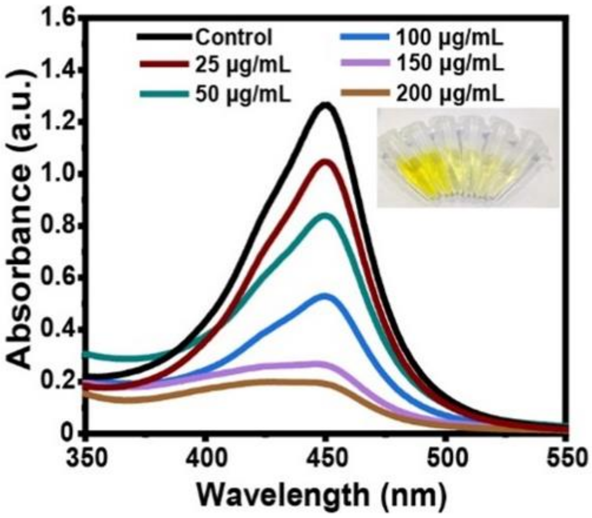

Figure S13. UV–vis spectra of TMB solution in the presence of H_2O_2 and different concentrations of MnO_2-Au-mSiO_2@Cur with stopping solution (1% H_2SO_4).

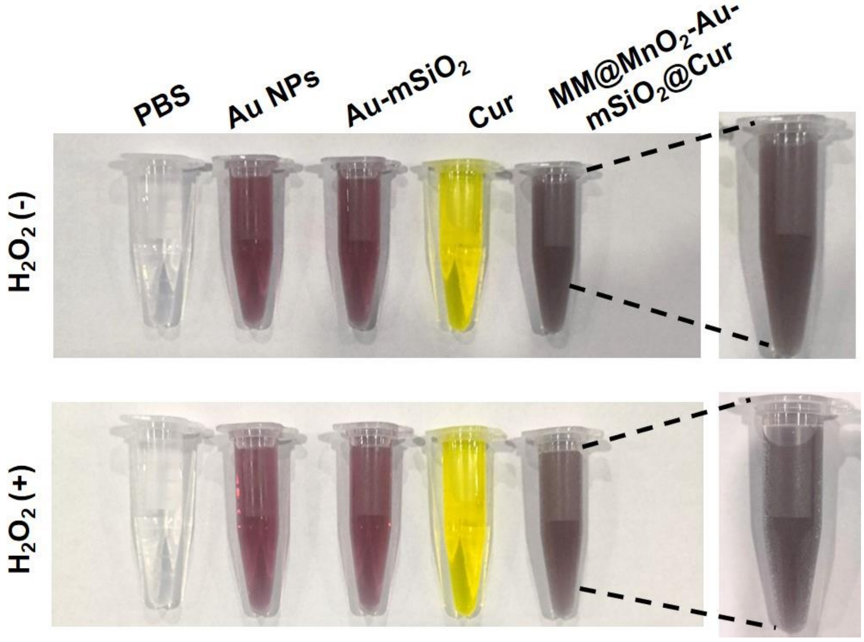

Figure S14. Photographs of different samples with or without incubation with 1 mM H_2O_2.

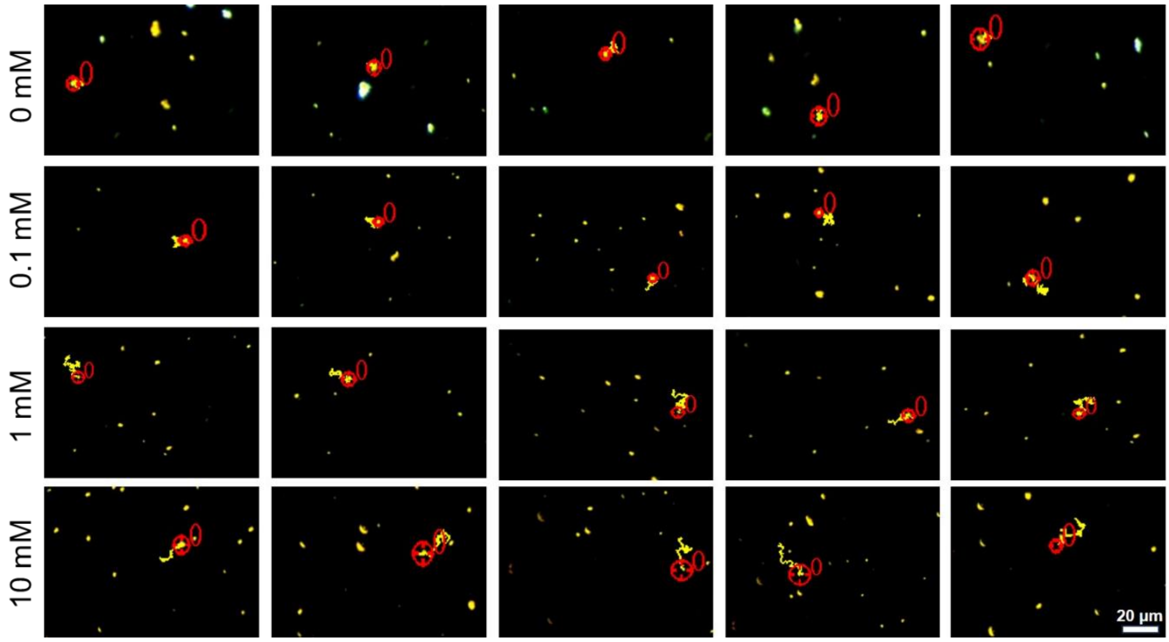

Figure S15. Motion trajectories of the nanomotors after treatment with different concentrations of H_2O_2. Scale bar, 20 µm.

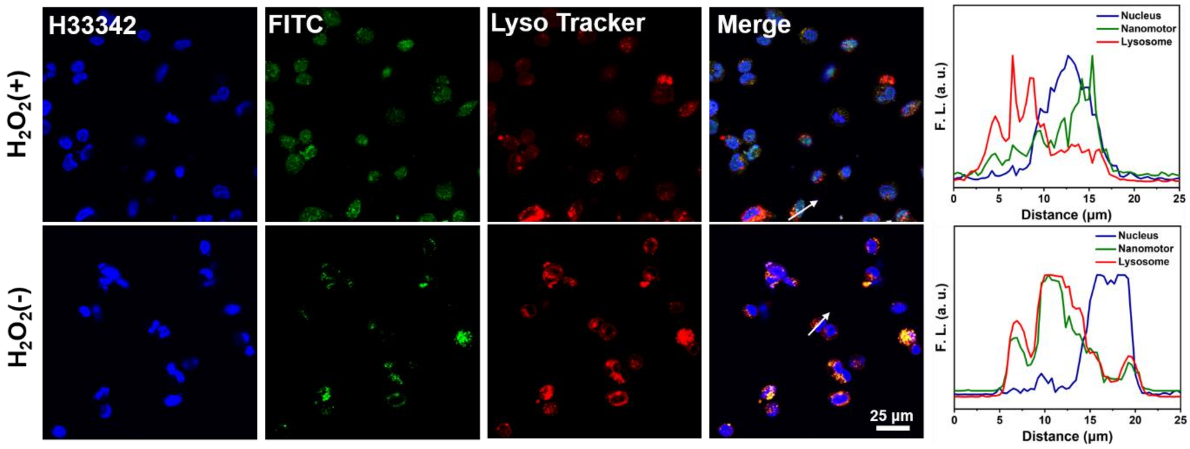

Figure S16. LysoTracker staining of MnO_2-Au-$mSiO_2$ nanoparticles with or without H_2O_2 treatment.

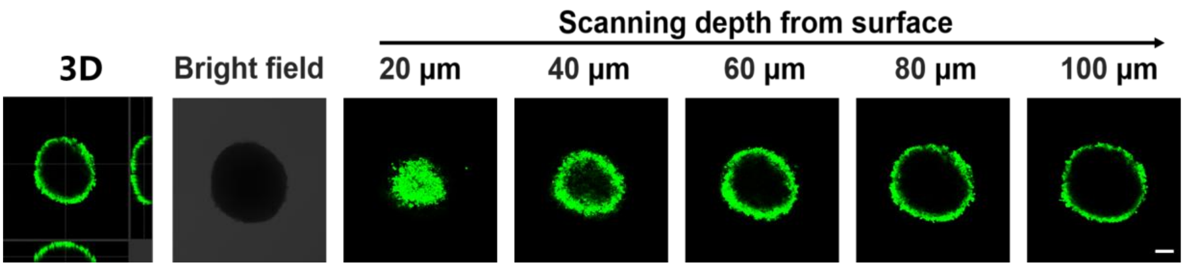

Figure S17. CLSM images of 3D cell spheroids treated with FITC-labeled Au-mSiO_2 under H_2O_2 stimulation.

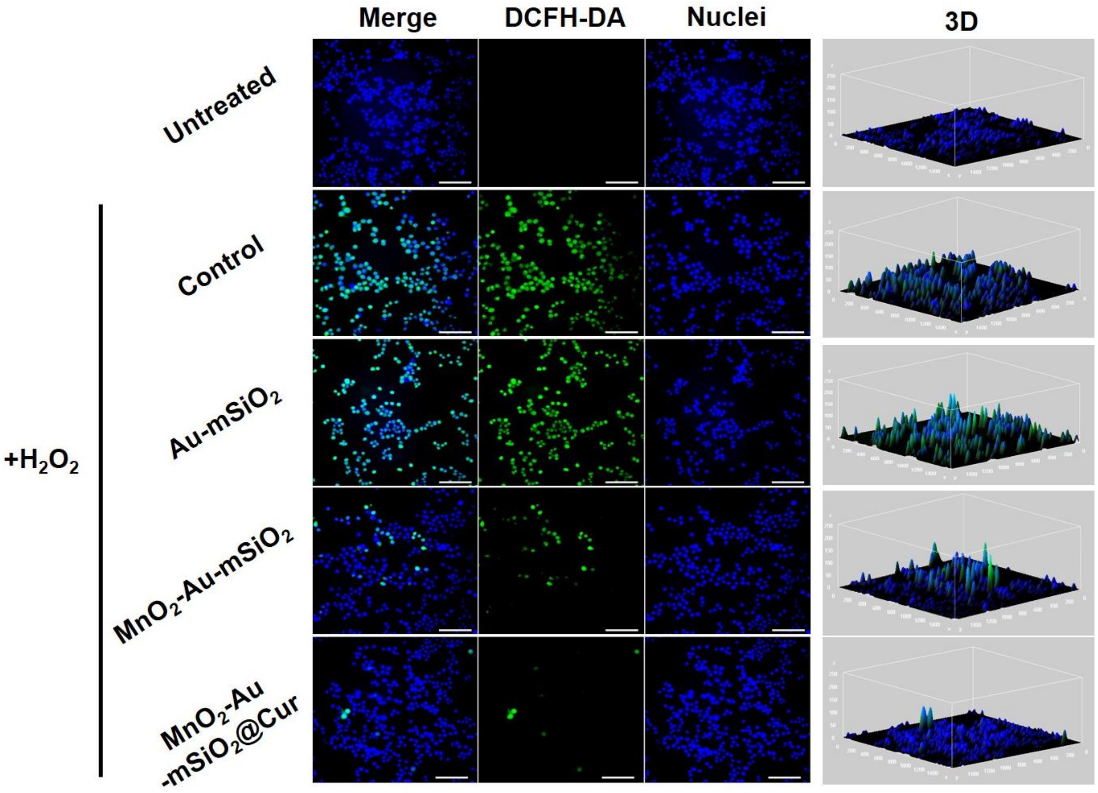

Figure S18. Fluorescence images of cellular ROS levels in BV2 cells after different treatments. Scale bar, 100 μm.

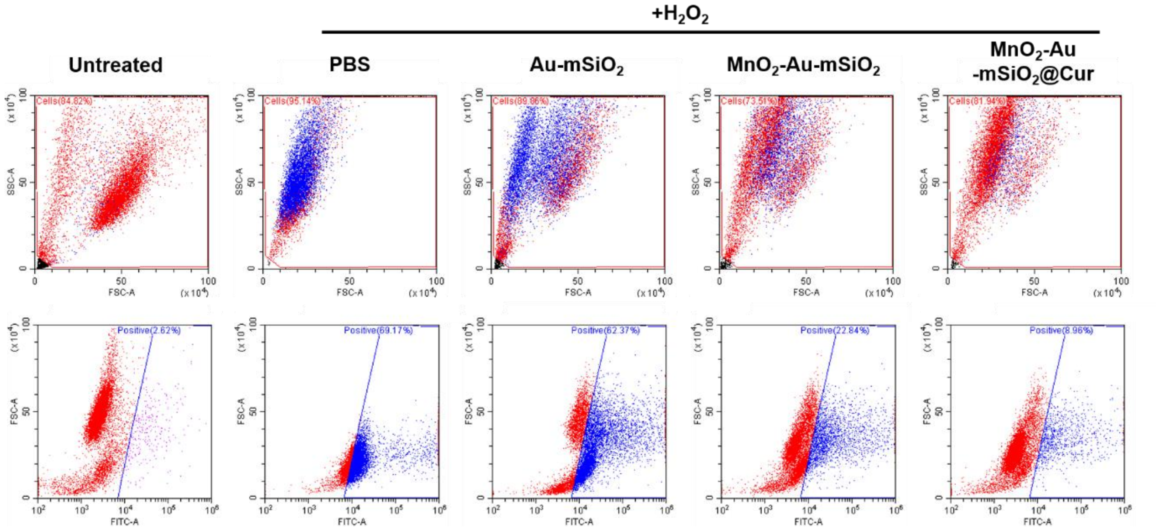

Figure S19. FCM images of SH-SY5Y cells stained with an intracellular ROS kit after different treatments. These flow cytometry gating strategies correspond to the experiments shown in Figure 5E.

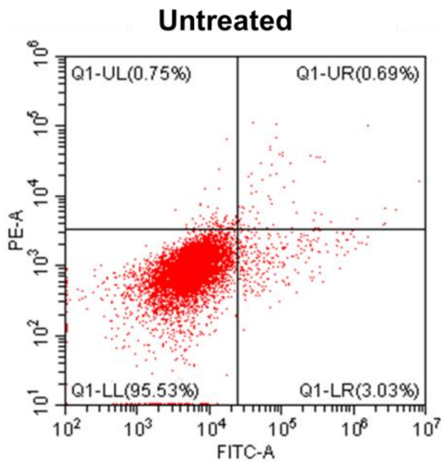

Figure S20. FCM analysis of SH-SY5Y cells not treated with H2O2 (as a control group) was performed via Annexin V-FITC/PI staining.

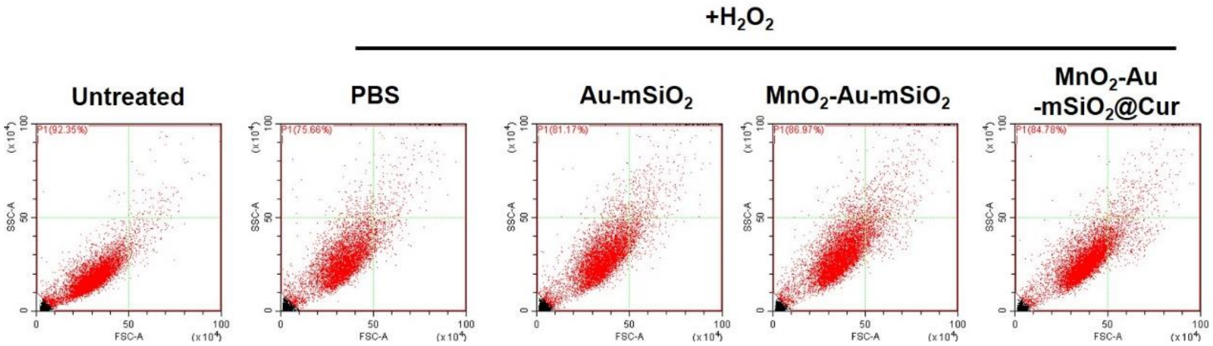

Figure S21. FCM images of SH-SY5Y cells stained with an Annexin V-FITC/PI kit after different treatments. These flow cytometry gating strategies correspond to the experiments shown in Figure 5G and Figure S20.

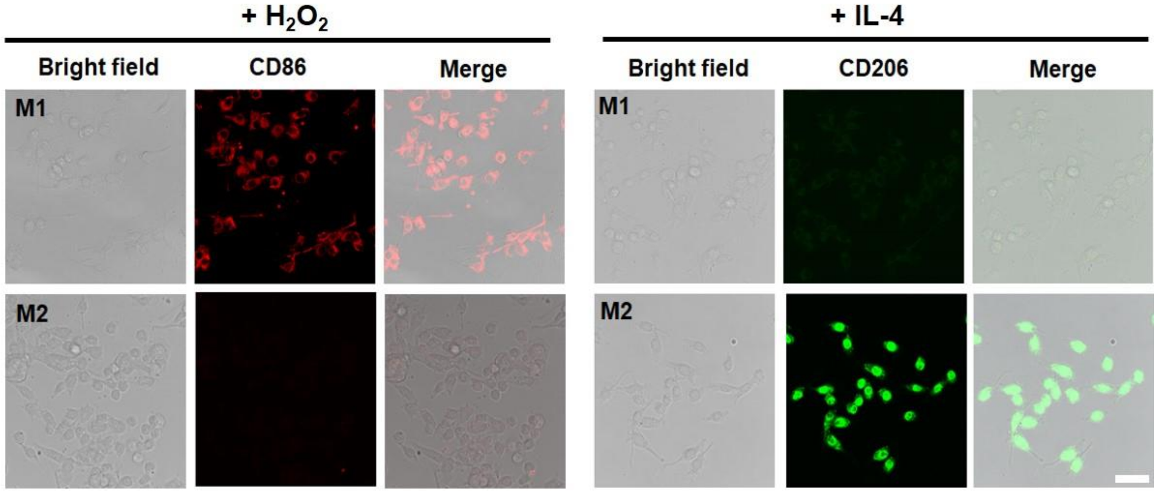

Figure S22. CLSM images of BV2 cells after H_2O_2 and IL-4 treatments. Scale bar, 20 μm.

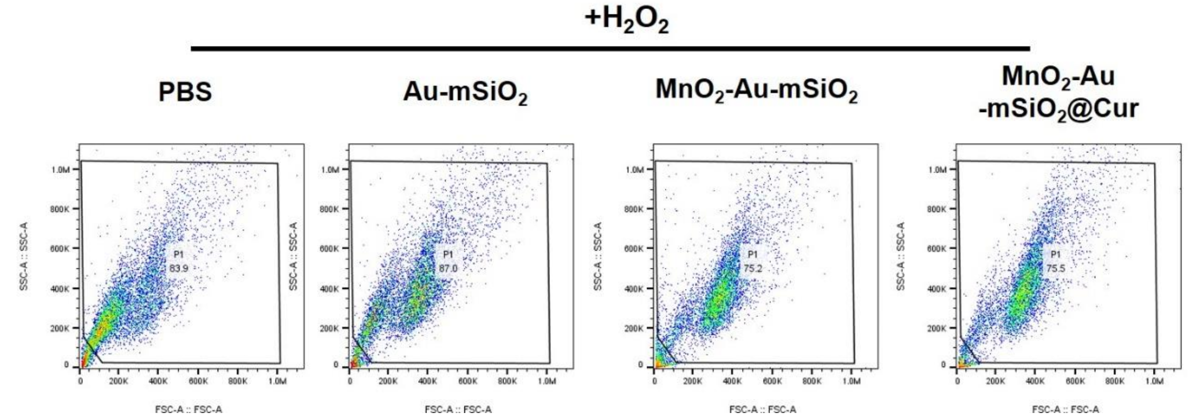

Figure S23. FCM images of BV2 cells stained with CD86/CD206 after different treatments. These flow cytometry gating strategies correspond to the experiments shown in Figure 6I.

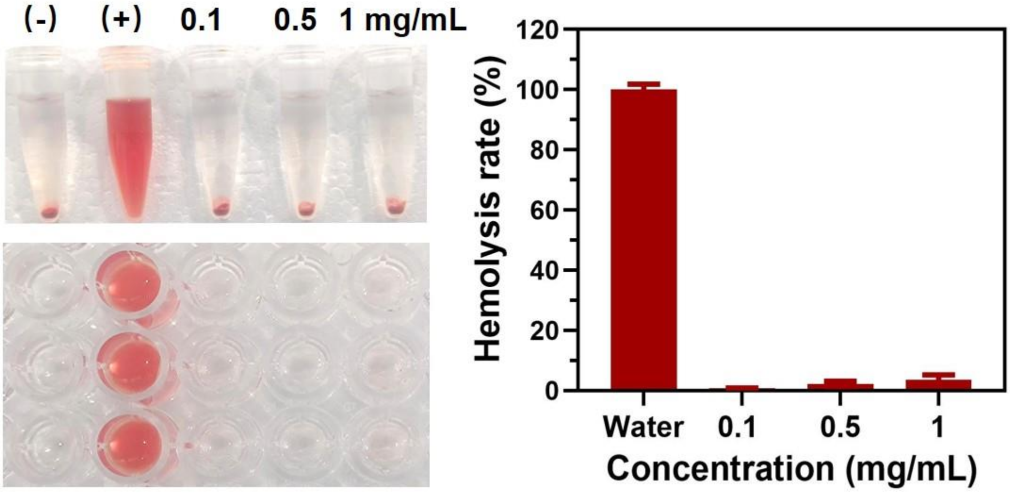

Figure S24. Hemolysis tests of MM@MnO$_2$-Au-mSiO$_2$@Cur at different concentrations.

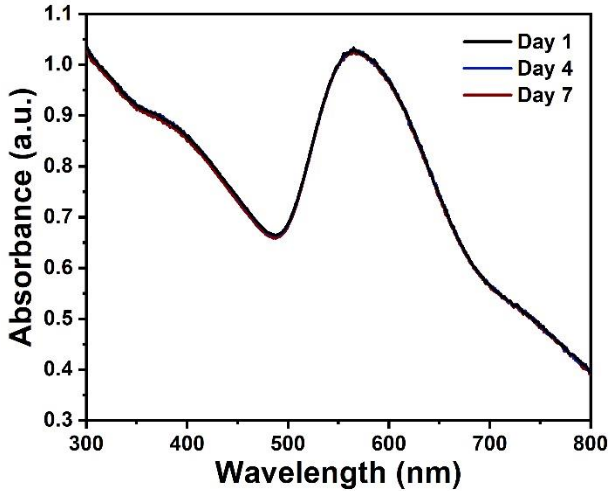

Figure S25. UV–vis spectrum of MM@MnO_2-Au-$mSiO_2$@Cur coincubated with PBS for 7 days.

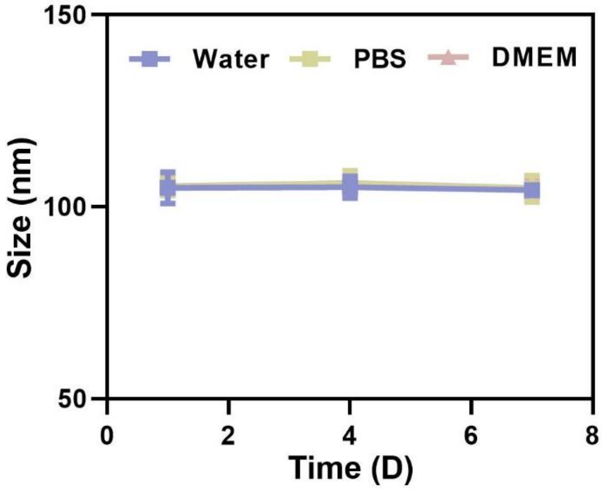

Figure S26. The stability of MM@MnO_2-Au-$mSiO_2$@Cur after coincubated with water, PBS, or DMEM detected by DLS.

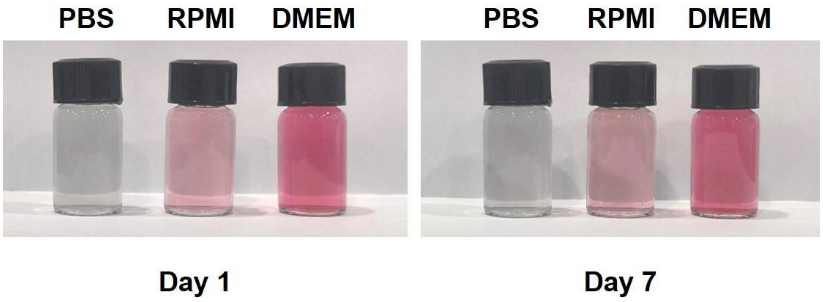

Figure S27. The photographs of MM@MnO_2-Au-mSiO_2@Cur nanomotors on day 1 and day 7 after incubated with PBS, RPMI, and DMEM solution.

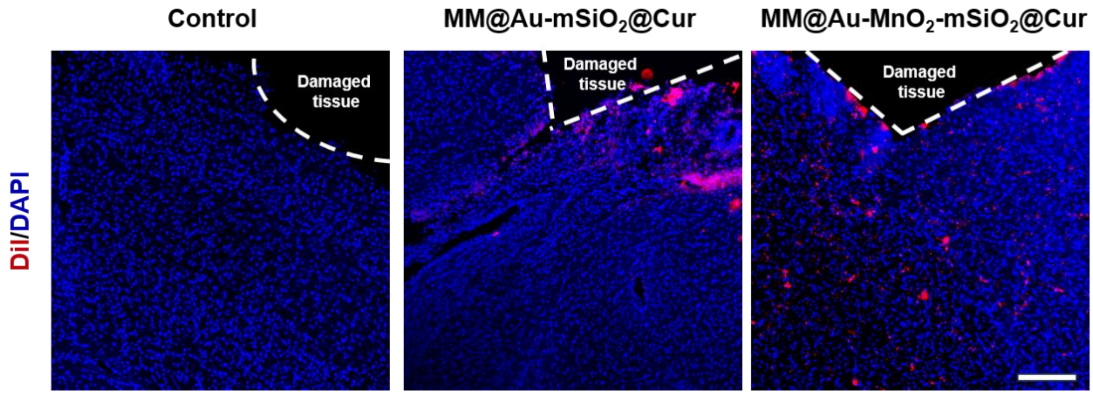

Figure S28. The CLSM images of the brain slice of TBI mice after different treatments. Scale bar = 200 μm.

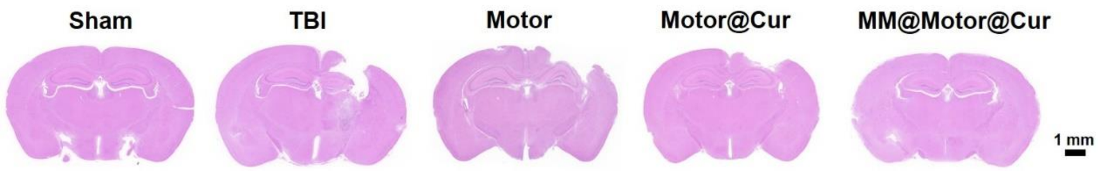

Figure S29. H&E staining of representative brain tissue 14 days after treatment.

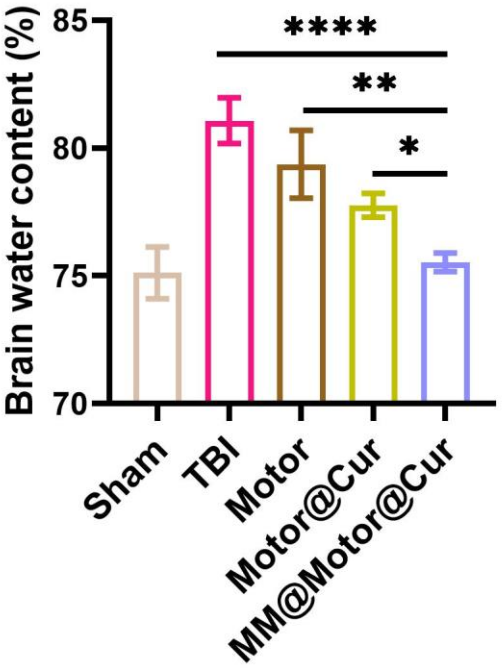

Figure S30. Water content in brain tissue after different treatments. The results are presented as the mean ± SD (n = 3). Significant differences were assessed by one-way ANOVA (*P <0.05, **P <0.01, ****P < 0.0001).

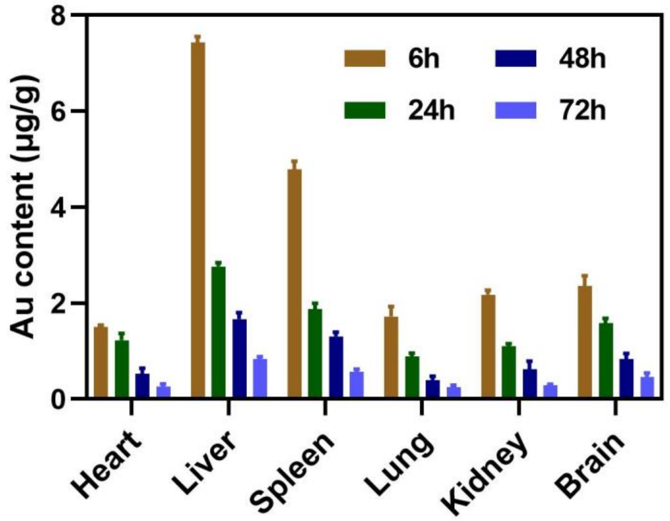

Figure S31. Biodistribution of the biomimetic nanomotors at different times after injection.

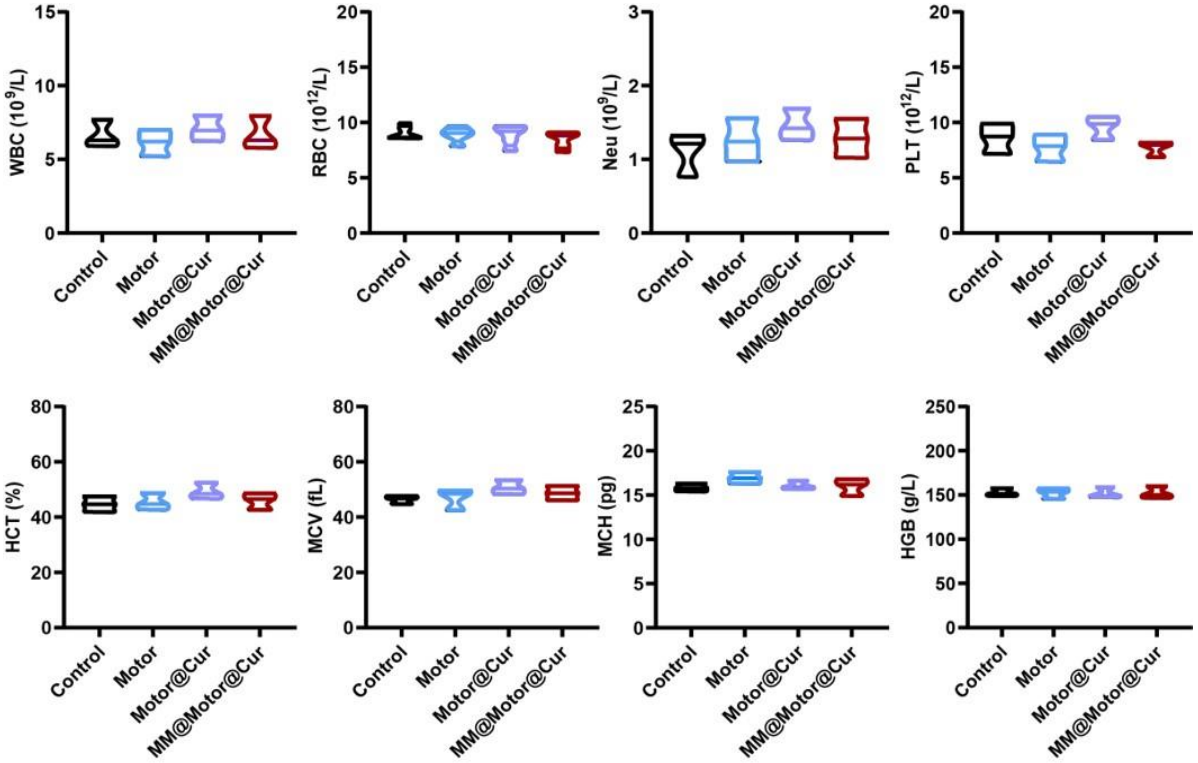

Figure S32. Blood hematology analysis of mice under different treatments.

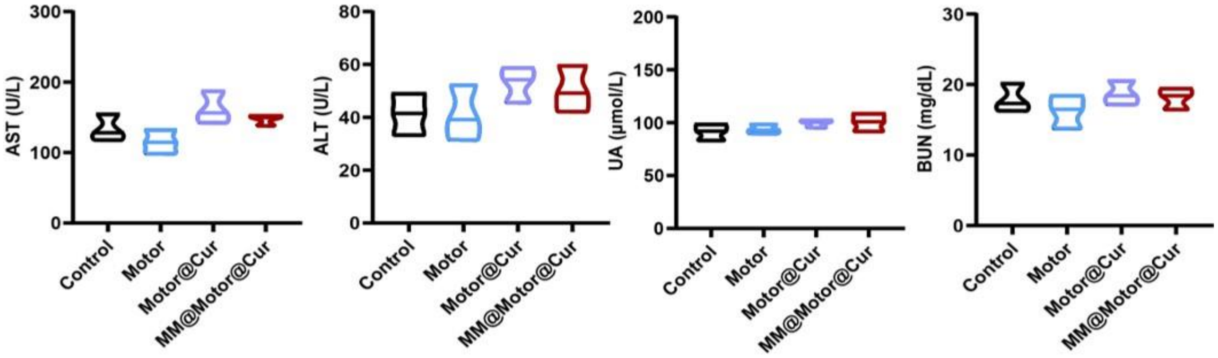

Figure S33. Blood biochemical analysis of liver and kidney function in mice after different treatments.

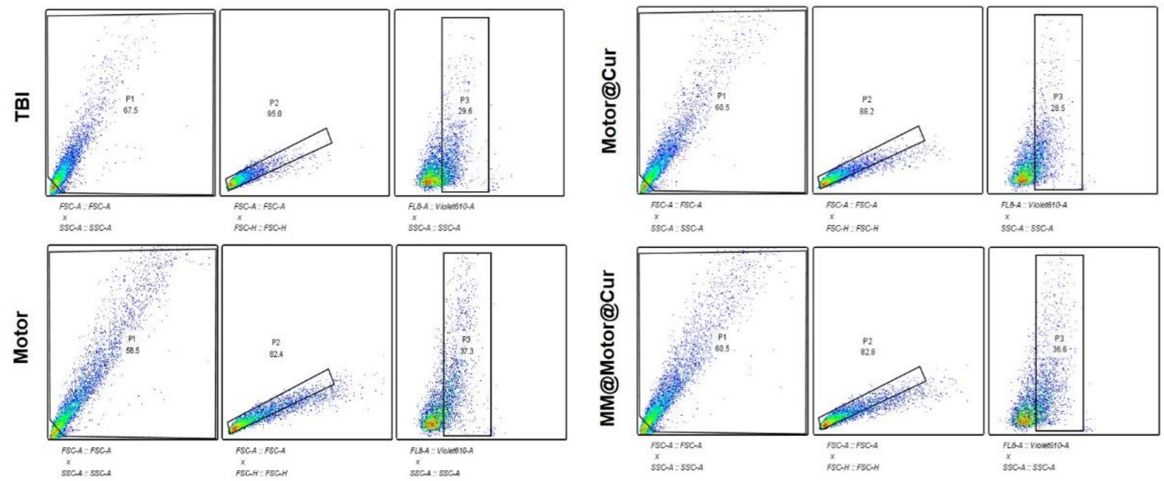

Figure S34. FCM images of brain tissues from TBI mice after different treatments. The flow cytometry gating strategies used for the experiments are shown in Figure 8G.
